# Targeting β2‐Adrenergic Receptor Stability With Lycorine Reveals a Host‐Directed Pathway for Antiviral Defense Against Poxviruses

**DOI:** 10.1002/advs.77306

**Published:** 2026-08-21

**Authors:** Xin Zhang, Shuqin Xu, Zixuan Zhang, Chenwen Wan, Xiaoqin Wang, Peihua Wang, Huini Yang, Tianxiao Huang, Yijia Yuan, Faiza Atique, Rui Yang, Yingli He, Zhijun Liu

**Affiliations:** ^1^ Department of Infectious Diseases State Key Laboratory for Diagnosis and Treatment of Severe Zoonotic Infectious Diseases The First Affiliated Hospital of Xi'an Jiaotong University Xi'an Shaanxi China; ^2^ Department of Hepatobiliary Surgery The First Affiliated Hospital of Xi'an Jiaotong University Xi'an Shaanxi China; ^3^ National Local Joint Engineering Research Center for Precision Surgery & Regenerative Medicine the First Affiliated Hospital of Xi'an Jiaotong University Xi'an China; ^4^ Institute of Neuroscience Translational Medicine Institute School of Basic Medical Sciences Health Science Center Xi'an Jiaotong University Xi'an China; ^5^ School of Future Technology Xi'an Jiaotong University Xi'an China

**Keywords:** host‐directed antiviral therapy, lycorine, poxvirus, psychological stress, β2‐AR stabilization

## Abstract

Psychological stress is a pervasive yet poorly understood modulator of infectious disease outcomes. Here, we report a mechanistic link between chronic stress and severe viral pathogenesis, identifying the β2‐adrenergic receptor (β2‐AR) as a critical host factor and therapeutic target. Using a model of chronic restraint stress and cowpox virus (CPXV) infection, we demonstrate that psychological stress significantly exacerbates disease severity and mortality. This increased susceptibility is driven by autophagy‐mediated downregulation of β2‐AR, a process that is recapitulated during viral infection. Accordingly, genetic loss‐of‐function and pharmacological approaches established β2‐AR as a critical host protective factor that suppresses CPXV replication. Leveraging this pathway, we identify the natural alkaloid lycorine as a novel β2‐AR‐stabilizing ligand that rescues the receptor from degradation. Lycorine exhibits potent antiviral activity by suppressing post‐entry viral replication. In vivo, lycorine reduced viral burden and tissue pathology and improved survival following CPXV infection, and these protective effects required cluster of differentiation 8‐positive (CD8^+^) T cells. Mechanistically, lycorine attenuates virus‐induced TANK‐ binding kinase 1/interferon regulatory factor 3 (TBK1/IRF3)associated inflammatory signaling while enhancing antiviral Interferon (IFN)/interferon‐stimulated gene (ISG) responses through β2‐AR‐associated and additional stimulator of interferon gene (STING)‐independent regulatory mechanisms. Collectively, our findings reveal β2‐AR as a molecular link between psychological stress and antiviral immunity and highlight lycorine‐mediated β2‐AR stabilization as a potential host‐directed antiviral strategy for poxvirus infection.

## Graphical abstract

Chronic stress drives autophagy‐mediated β2‐AR degradation, impairing antiviral immunity and promoting poxvirus lethality. Lycorine directly stabilizes β2‐AR to restore host defense, suppress excessive inflammation and improve survival.

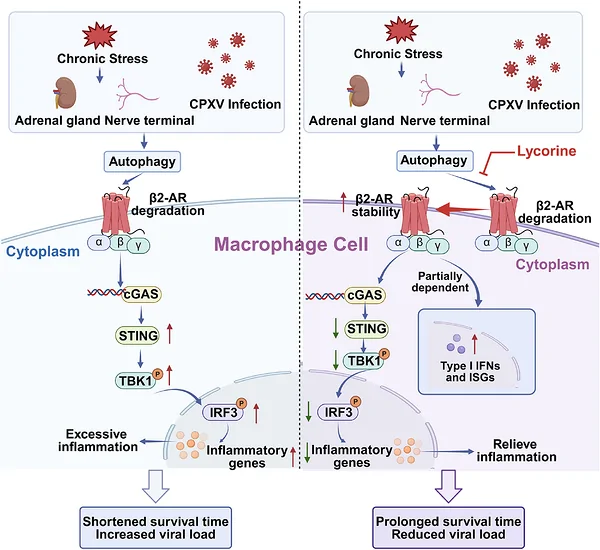

## 1 Introduction

Poxviruses of the genus Orthopoxvirus (OPXV), such as vaccinia virus (VACV), cowpox virus (CPXV), and monkeypox virus (MPXV), are large, cytoplasmic DNA viruses. Although smallpox has been eradicated, OPXVs remain a significant threat. This was starkly demonstrated in 2022, when two human‐adapted MPXV strains sparked a global outbreak across 115 non‐endemic countries [1, 2, 3]. From July 2022 to May 2023, the World Health Organization declared mpox a Public Health Emergency of International Concern (PHEIC) [4, 5]. This event resulted in over 99 000 cases globally, including more than 33 000 infections and 60 deaths in the United States [6, 7]. As of 2024, transmission persists, with approximately 200 new monthly cases still being reported in the U.S [8], underscoring the ongoing public health challenge posed by poxviruses. Despite the unique epidemiological features of the recent MPXV outbreak, its core biological processes, including genome replication and immune evasion, are highly conserved with prototypical OPXVs like VACV [9, 10, 11]. This evolutionary conservation validates the use of CPXV and VACV as model systems to elucidate fundamental mechanisms of poxvirus‐host interaction, providing critical insights relevant to MPXV pathogenesis. Currently, tecovirimat is the most widely used antiviral drug against mpox. It inhibits viral egress by binding to the viral phospholipase F13 protein. F13 forms a homodimer and that tecovirimat promotes dimerization to exert its antiviral effect [12]. Notably, mutations identified in clinical MPXV isolates are frequently located at the F13 dimer interface and impair drug‐induced dimerization [12], suggesting that MPXV can develop resistance to tecovirimat. This phenomenon not only limits the long‐term application of existing antivirals but also highlights the urgent need to develop novel therapeutic strategies, particularly those targeting host factors.

Psychological stress is increasingly recognized as an important determinant of susceptibility to infectious diseases [13, 14, 15]. A key mediator of this effect is the sympathetic nervous system, which acts on the downstream effector, the adrenal medulla, via neurotransmitters released from postganglionic fibers during chronic stress. The adrenal medulla then releases catecholamines such as epinephrine and norepinephrine into the bloodstream to exert their effects. The β2‐adrenergic receptor (β2‐AR), a G protein‐coupled receptor expressed on various immune cells including macrophages, is the primary sensor for these catecholamines and plays a central role in modulating cytokine secretion and antiviral immune responses [16, 17, 18, 19, 20]. Recent studies have further established β2‐AR as a critical regulator of antiviral immunity, wherein β2‐AR signaling controls early transcriptional programs during CD8^+^ T cell responses to viral infection involving over 300 genes related to cytokine receptor activation, neurotransmitter pathways, and circadian rhythms [21], whereas β2‐AR directly entrains circadian gene oscillation and CD8^+^ T cell differentiation in response to virus infection [22], and additionally β2‐AR signaling downregulates innate immune responses by suppressing NK cell IFN‐γ production, thereby reducing host resistance to viral infection [19]. Notably, our preliminary data reveal that chronic stress promotes autophagy‐dependent β2‐AR downregulation, thereby exacerbating disease severity and mortality upon cowpox virus (CPXV) challenge, and that CPXV itself actively downregulates β2‐AR as an immune evasion strategy. More importantly, the functional outcomes of β2‐AR activation are highly context‐dependent. For example, in the cardiovascular system, the β2‐AR‐PI3K/AKT signaling axis promotes cardiomyocyte survival [23]. In contrast, within the tumor microenvironment, such as in nicotine‐induced hepatocellular carcinoma, this same cascade can accelerate tumor proliferation and metastasis [24]. This functional duality suggests that β2‐AR signaling may also play a complex and nuanced role during poxvirus infection, a possibility that warrants further mechanistic investigation.

Lycorine, a natural alkaloid derived from plants of the Amaryllidaceae family, possesses broad‐spectrum antiviral and immunomodulatory activities against numerous RNA viruses [25, 26, 27]. Poxviruses evade host immunity by targeting key innate immune pathways, including cyclic GMP‐AMP synthase (cGAS)‐stimulator of interferon genes (STING) signaling, while also exploiting the PI3K/AKT pathway to promote viral replication and immune evasion [28, 29]. These observations raise the possibility that lycorine may exert anti‐poxviral activity against orthopoxviruses by modulating these host signaling pathways.

In this study, we identify a previously unrecognized neuroimmune mechanism linking psychological stress, β2‐AR regulation, and orthopoxvirus pathogenesis. We demonstrate that orthopoxvirus infection induces β2‐AR degradation, thereby impairing host antiviral defense and promoting viral replication. Through small molecule screening, we identify lycorine as a natural β2‐AR‐stabilizing compound that restores receptor expression and protects against poxvirus infection. Mechanistically, lycorine suppresses excessive inflammatory activation while enhancing antiviral IFN/ISG responses through β2‐AR‐associated and additional regulatory mechanisms. Furthermore, we demonstrate that the protective effects of lycorine require myeloid β2‐AR and are mediated predominantly through CD8^+^ T cell‐dependent immunity. Together, our findings identify β2‐AR as an important host protective factor linking psychological stress to antiviral immunity and establish β2‐AR stabilization as a potential host‐targeted therapeutic strategy against poxvirus infection.

## 2 Results

### 2.1 Chronic Stress Exacerbates CPXV Infection and Mortality In Vivo

**FIGURE 1:**
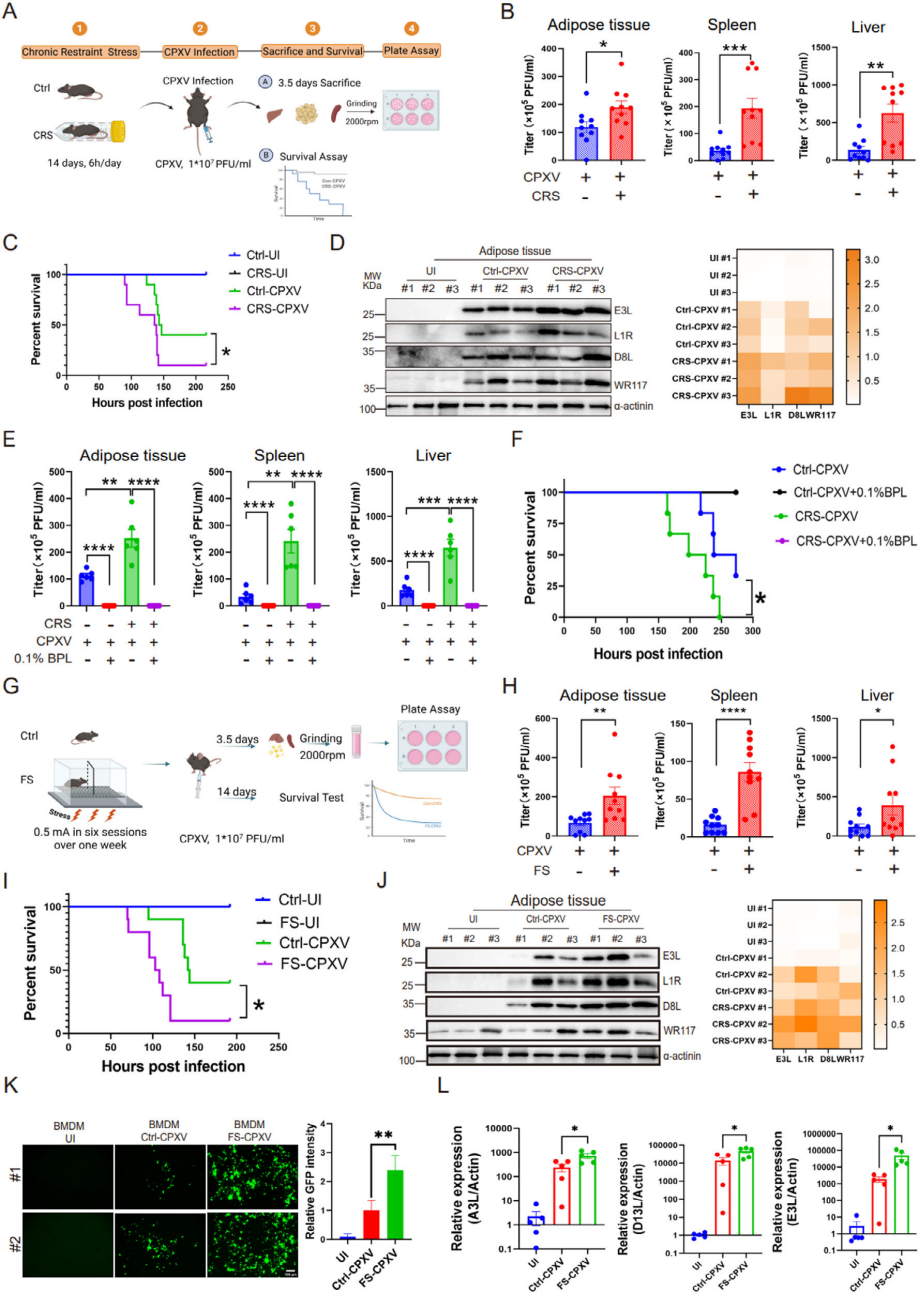
Chronic stress exacerbates CPXV infection and lethality in vivo. (A) Schematic of the CRS model and CPXV infection. C57/BL6 mice were subjected to CRS (6 h/day for 14 days), followed by CPXV infection (1 × 10^7^ pfu). (B) Viral titers in epididymal adipose tissue, spleen and liver at 3.5 dpi (*n* =10/group). (C) Survival of control and CRS mice following CPXV infection (*n* =10/group). Statistical significance was determined by the log‐rank (Mantel–Cox) test. (D) Immunoblot analysis of viral proteins (E3L, L1R, D8L, WR117) in adipose tissue (*n* =3/group). (E) Viral titers in epididymal adipose tissue, spleen and liver from mice infected with untreated or 0.1% BPL‐inactivated CPXV at 3.5 dpi (*n* =6/group). (F) Survival of control and CRS mice following infection with untreated or BPL‐inactivated CPXV (*n* =6/group). Statistical significance was determined by the log‐rank (Mantel–Cox) test. (G) Schematic of the FS model and CPXV infection. C57BL/6 mice received foot shock (0.5 mA, six times/day) for 7 days before CPXV infection. (H) Viral titers in epididymal adipose tissue, spleen and liver at 3.5 dpi (*n* =10/group). (I) Survival of control and FS mice following CPXV infection (*n* =10/group). Statistical significance was determined by the log‐rank (Mantel‐Cox) test. (J) Immunoblot analysis of viral proteins (E3L, L1R, D8L, WR117) in adipose tissue (*n* =3/group). (K) Representative GFP fluorescence images of BMDMs isolated from CPXV‐infected control and FS mice. (L) qRT‐PCR analysis of viral genes (A3L, E3L, D13L) in BMDMs. Data are presented as mean ± SEM. Individual data points represent biologically independent samples, and n represents the number of biologically independent animals as detailed above. Statistical significance was determined using unpaired two‐tailed Student's *t*‐test or log‐rank (Mantel–Cox) test. ^*^ *p* < 0.05, ^**^ *p* < 0.01, ^***^ *p* < 0.001, ^****^ *p* < 0.0001; *ns*, not significant.

Psychological stress is known to impair innate immunity [30] and increase susceptibility to viral infections [31]. To investigate the potential impact of stress and anxiety on orthopoxvirus infection, we established two stress models: chronic restraint stress (CRS) (Figure 1A) and foot shock (FS) prior to CPXV challenge (Figure 1G). Consistent with previous reports, CRS increased circulating corticosterone and norepinephrine levels (Figure S1A). FS‐induced stress was confirmed by elevated plus maze and open field tests, which demonstrated increased anxiety‐like behaviors and reduced exploratory activity (Figure S1B–E). These results verified the successful establishment of both stress paradigms.

To determine the effect of stress on viral susceptibility, viral burdens were assessed in the liver, spleen and epididymal adipose tissues at 3.5 days post‐infection (dpi). CRS markedly increased viral titers in all examined tissues and significantly reduced survival compared with control mice (Figure 1B,C). Consistently, expression of the viral proteins E3L, L1R, D8L and WR117 was substantially elevated in adipose tissues from stressed animals (Figure 1D). Moreover, BMDMs isolated from CRS‐treated mice exhibited enhanced CPXV replication, as evidenced by increased GFP signal, viral protein abundance, and viral gene expression (A3L, D13L and E3L) (Figure S1F–H). These findings indicate that chronic stress promotes CPXV replication and dissemination, potentially through altered macrophage antiviral responses.

To distinguish the relative contributions of viral replication and virus‐induced inflammation to stress‐associated mortality, CPXV was inactivated with 0.1% β‑propiolactone (BPL). Although BPL‐inactivated CPXV retained its ability to induce inflammatory responses, it completely lost replicative capacity (Figure S1I). Under these conditions, all mice survived and no infectious virus was detected, irrespective of stress exposure. In contrast, CRS significantly increased viral loads and mortality in mice infected with replication‐competent CPXV (Figure 1E,F). These results indicate that enhanced mortality under stress is primarily driven by increased viral replication rather than by inflammatory stimulation alone. Importantly, the effects observed in the CRS model were fully recapitulated in an independent FS stress model, including elevated viral burdens and reduced survival (Figure 1H–L and Figure S1J), indicating that distinct forms of psychological stress converge on a common mechanism that enhances poxvirus infection.

To further examine the influence of environmental factors on antiviral immunity, we employed an enriched environment (EE) model, a well‐established intervention that mitigates depressive‐like behaviors (Figure S2A). Behavioral analyses confirmed reduced anxiety and enhanced exploratory activity in EE mice compared with standard environment (SE) controls (Figure S2B–E). Notably, EE housing significantly reduced viral replication and improved resistance to CPXV infection (Figure S2F–I). Thus, environmental enrichment exerts effects opposite to those of psychological stress, highlighting the profound influence of environmental cues on host antiviral immunity.

In conclusion, these findings demonstrate that chronic psychological stress enhances CPXV replication, dissemination and mortality, whereas environmental enrichment confers significant antiviral protection, underscoring a critical role for neuroimmune regulation in determining host susceptibility to poxvirus infection.

### 2.2 Autophagy‐Mediated Downregulation of β2‐AR Underlies Stress‐Exacerbated Viral Pathogenesis

**FIGURE 2:**
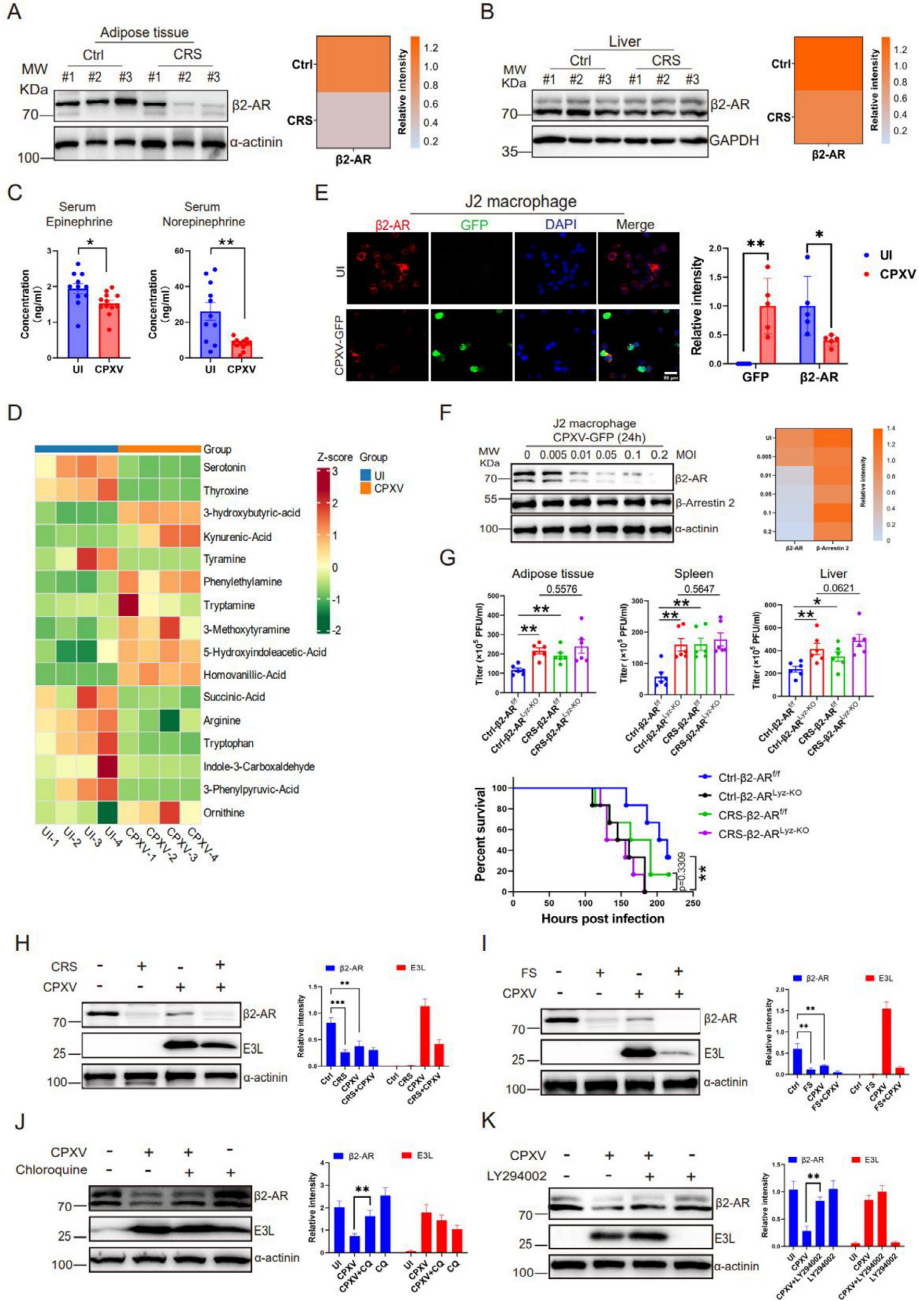
Autophagy‐mediated downregulation of β2‐AR underlies stress‐exacerbated viral pathogenesis. (A,B) Immunoblot analysis of β2‐AR in epididymal adipose tissue and liver of mice subjected to CRS. (C) Measurement of epinephrine and norepinephrine in the serum of CPXV‐infected mice by ELISA (*n* = 11∼12/group). (D) Targeted metabolomic analysis of stress hormones in the serum of CPXV‐infected mice. C57BL/6 male mice (*n* = 5/group) were uninfected or intraperitoneally infected with CPXV (1 × 10^7^ pfu), and at the indicated times, the serum was collected and target metabolomic analysis was performed by liquid chromatography‐mass spectrometry (LC‐MS). (E) Immunostaining assay was used to analyze the localization of β2‐AR (red) and CPXV (green) in J2 macrophages. Scale bars, 50 µm. (F) β2‐AR expression is modulated in an MOI‐dependent manner. The J2 macrophages were infected with CPXV for the indicated multiplicities before immunoblot analysis. (G) Viral titers and survival analysis of CPXV‑infected β2‐AR^f/f^ and β2‐AR^Lyz‐KO^ mice subjected to CRS (*n* = 6/group). Statistical significance was determined by the log‐rank (Mantel‐Cox) test. (H,I) β2‐AR protein expression in BMDMs from control, CRS (H) and FS (I) groups treated with or without CPXV infection. (J,K) Effects of autophagy inhibition on CPXV‐induced β2‐AR downregulation. J2 macrophages (1 × 10^6^ cells) were pretreated with Chloroquine (10 µm) (J) or LY294002 (2.5 µm) (K) for 2 h, followed by CPXV infection (MOI = 0.1) for 24 h in the continued presence of the inhibitors. Similar results were obtained from three biologically independent experiments (C,D, H,K). Data are presented as mean ± SEM. Individual data points represent biologically independent samples, and n represents the number of biologically independent animals as detailed above. Statistical analysis was determined using the unpaired two‐tailed *t*‐test. ^*^ *p* < 0.05, ^**^ *p* < 0.01, ^***^ *p* < 0.001, ^****^ *p* < 0.0001; *ns*, not significant.

Chronic psychological stress activates the sympathetic nervous system and elevates catecholamine release, which signals through β2‐adrenergic receptors (β2‐AR) expressed on immune cells [32, 33]. Previous studies have shown that sustained catecholamine stimulation promotes trafficking of activated β2‐AR to autophagosomes, resulting in lysosomal degradation and prolonged receptor desensitization [34]. Consistent with this mechanism, CRS markedly reduced β2‐AR expression in epididymal adipose tissue, liver and BMDMs (Figure 2A,B and Figure S3A), suggesting that chronic stress disrupts β2‐AR signaling and may compromise antiviral immunity [35, 36]. To investigate whether CPXV infection also perturbs neuroendocrine signaling, we measured circulating catecholamine levels following infection. Unexpectedly, both epinephrine (EPI) and norepinephrine (NE) were significantly decreased at 3.5 dpi (Figure 2C). Metabolomic analysis further revealed broad alterations in catecholamine‐associated metabolic pathways and precursor metabolites (Figure 2D), suggesting that the CPXV infection induces substantial neuroendocrine remodeling.

Because stress‐induced β2‐AR downregulation was associated with enhanced viral susceptibility [18], we next examined whether CPXV directly regulates β2‐AR expression. J2 and RAW264.7 macrophages were infected with CPXV‐GFP at increasing MOIs or harvested at different time points post‐infection. CPXV infection induced a dose‐ and time‐dependent reduction in β2‐AR protein expression, whereas the levels of its adaptor protein β‐arrestin2 remained unchanged (Figure 2F and Figure S3B–E). Immunofluorescence analysis further confirmed a marked loss of β2‐AR staining in infected cells (Figure 2E and Figure S3F). These findings were further validated in CPXV‐infected BMDMs isolated at 24, 48, 72, and 96 h post‐infection (Figure S3G). Both β2‐AR protein and mRNA levels progressively declined following infection (Figure S3H,I).

To determine whether β2‐AR is required for stress‐induced enhancement on CPXV infection, we subjected β2‐AR knockout mice to CRS prior to CPXV infection. Notably, CRS failed to further increase viral loads and mortality in β2‐AR‐deficient mice compared with non‐stressed β2‐AR knockout controls (Figure 2G), indicating that β2‐AR is required for stress‐induced enhancement of CPXV infection. Moreover, prior exposure to CRS further enhanced CPXV‐induced β2‐AR downregulation (Figure 2H,I), suggesting that chronic stress preconditions host cells for heightened virus‐mediated suppression of β2‐AR signaling.

We next investigated the mechanism responsible for β2‐AR loss during infection. Pharmacological inhibition of autophagy with chloroquine (CQ), LY294002 and bafilomycin A1 effectively restored β2‐AR expression in CPXV‐infected cells (Figure 2J,K and Figure S3J). In contrast, the proteasome inhibitor MG132 failed to rescue β2‐AR levels (Figure S3K). Together, these findings demonstrate that CRS and CPXV infection converge on a common autophagy‐dependent mechanism that depletes β2‐AR, thereby providing a mechanistic basis for stress‐enhanced poxvirus pathogenesis.

### 2.3 Restoring β2‐AR Signaling Restricts CPXV Infection and Pathogenesis

**FIGURE 3:**
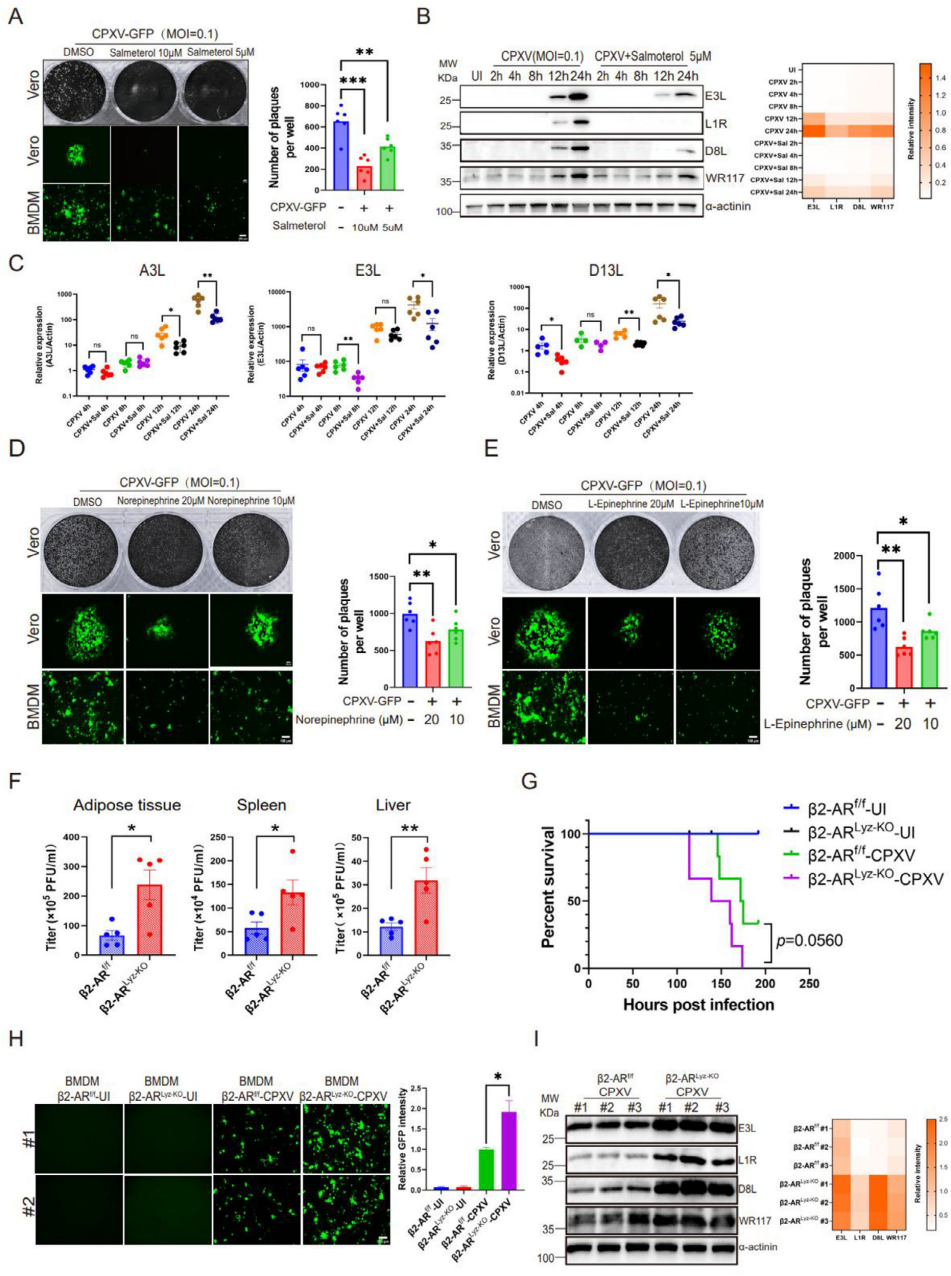
Restoring β2‐AR signaling restricts CPXV infection and pathogenesis. (A) Antiviral activity of Salmeterol (5 or 10 µm) against CPXV infection. Cytopathic effects were assessed at 48 h post‐infection, and viral infection was assessed by GFP fluorescence at 24 h. (B,C) Salmeterol inhibits viral proteins (B) and genes (C) in a time‐dependent manner. (D,E) Effects of other β2‐AR agonists on CPXV‐induced viral infection. (F) Titers in organs of β2‐AR myeloid KO mice. β2‐AR myeloid‐specific knockout male mice and their littermate controls were intraperitoneally injected with CPXV (1×10^7^ pfu). Target organs were harvested at 3.5 dpi, homogenized, and used to infect Vero cells for viral titer determination via plate assay (*n* =5/group). (G) Survival analysis of CPXV‐infected male mice with myeloid β2‐AR knockout. Survival was monitored over time for analysis (*n* =6/group). The survival rates were recorded daily for 2 weeks. Statistical significance was determined by the log‐rank (Mantel–Cox) test. (H) Representative GFP fluorescence images of BMDMs isolated from mock‐infected and CPXV infected β2‐AR^f/f^ and β2‐AR^Lyz‐KO^ mice. The intensity of GFP signal correlates with the level of viral infection. (I) Viral proteins (E3L, L1R, D8L, WR117) expression of BMDMs isolated from CPXV infected β2‐AR^f/f^ and β2‐AR^Lyz‐KO^ mice (*n* =3 per group). Similar results were obtained from three biologically independent experiments (A‐E). Data are presented as mean ± SEM. Individual data points represent biologically independent samples, and n represents the number of biologically independent animals as detailed above. Statistical analysis was determined using the unpaired two‐tailed *t*‐test. ^*^ *p* < 0.05, ^**^ *p* < 0.01, ^***^ *p* < 0.001, ^****^ *p* < 0.0001; *ns*, not significant.

Given that CPXV infection induces β2‐AR downregulation, we next investigated whether pharmacological activation of β2‐AR could restore antiviral signaling and restrict viral replication. CPXV‐infected Vero cells were treated with several β2‐AR agonists, including salmeterol, salbutamol, isoprenaline and eformoterol hemifumarate. Plaque assays and GFP fluorescence analysis demonstrated that all four agonists inhibited CPXV replication, with salmeterol exhibiting the strongest antiviral activity and a minimum effective concentration of 5 µm (Figure 3A and Figure S4A–D). These findings indicate that activation of β2‐AR signaling suppresses poxvirus replication despite infection‐induced receptor downregulation.

To further characterize the antiviral activity of salmeterol, viral gene and protein expression were assessed following treatment. Salmeterol markedly reduced the expression of multiple viral proteins, including E3L, L1R, D8L, and WR117, at different stages of infection (Figure 3B). Likewise, transcription of the viral genes A3L, E3L, and D13L was significantly suppressed (Figure 3C). Consistent with these findings, stimulation with the endogenous β‐adrenergic agonists norepinephrine and L‐epinephrine also reduced CPXV replication (Figure 3D,E). Together, these results demonstrate that activation of β2‐AR signaling exerts potent antiviral effects against CPXV by limiting viral gene expression and protein synthesis. Because β2‐AR signaling is also implicated in immune regulation, we next evaluated the effect of salmeterol on virus‐induced inflammatory responses. Although CPXV infection markedly increased cytokine and chemokine expression in macrophages, salmeterol did not significantly reduce inflammatory mediator production at 24 hpi (Figure S4E). These data suggest that the antiviral activity of salmeterol is largely independent of its classical anti‐inflammatory functions and is more closely associated with direct regulation of antiviral host responses.

To define the role of β2‐AR in vivo, myeloid‐specific β2‐AR knockout mice (β2‐AR^Lyz‐KO^) were generated and validated by qRT‐PCR (Figure S4F,G). Following CPXV challenge, β2‐AR‐deficient mice exhibited significantly higher viral burdens in multiple tissues and reduced survival compared with littermate controls (Figure 3F,G). Consistent with these findings, BMDMs isolated from β2‐AR^Lyz‐KO^ mice supported greater CPXV replication, accompanied by increased viral gene transcription and viral protein expression (Figure 3H,I and Figure S4H). These results establish myeloid cell‐intrinsic β2‐AR as a critical host restriction factor that limits poxvirus replication and disease progression.

Taken together, these findings demonstrate that restoration of β2‐AR signaling through pharmacological agonists suppresses CPXV replication, whereas genetic ablation of β2‐AR enhances viral susceptibility. Thus, β2‐AR represents a key molecular link between stress‐associated neuroendocrine signaling and antiviral host defense.

### 2.4 Identification of Lycorine as a β2‐AR‐Stabilizing Antiviral Compound Against CPXV Infection

**FIGURE 4:**
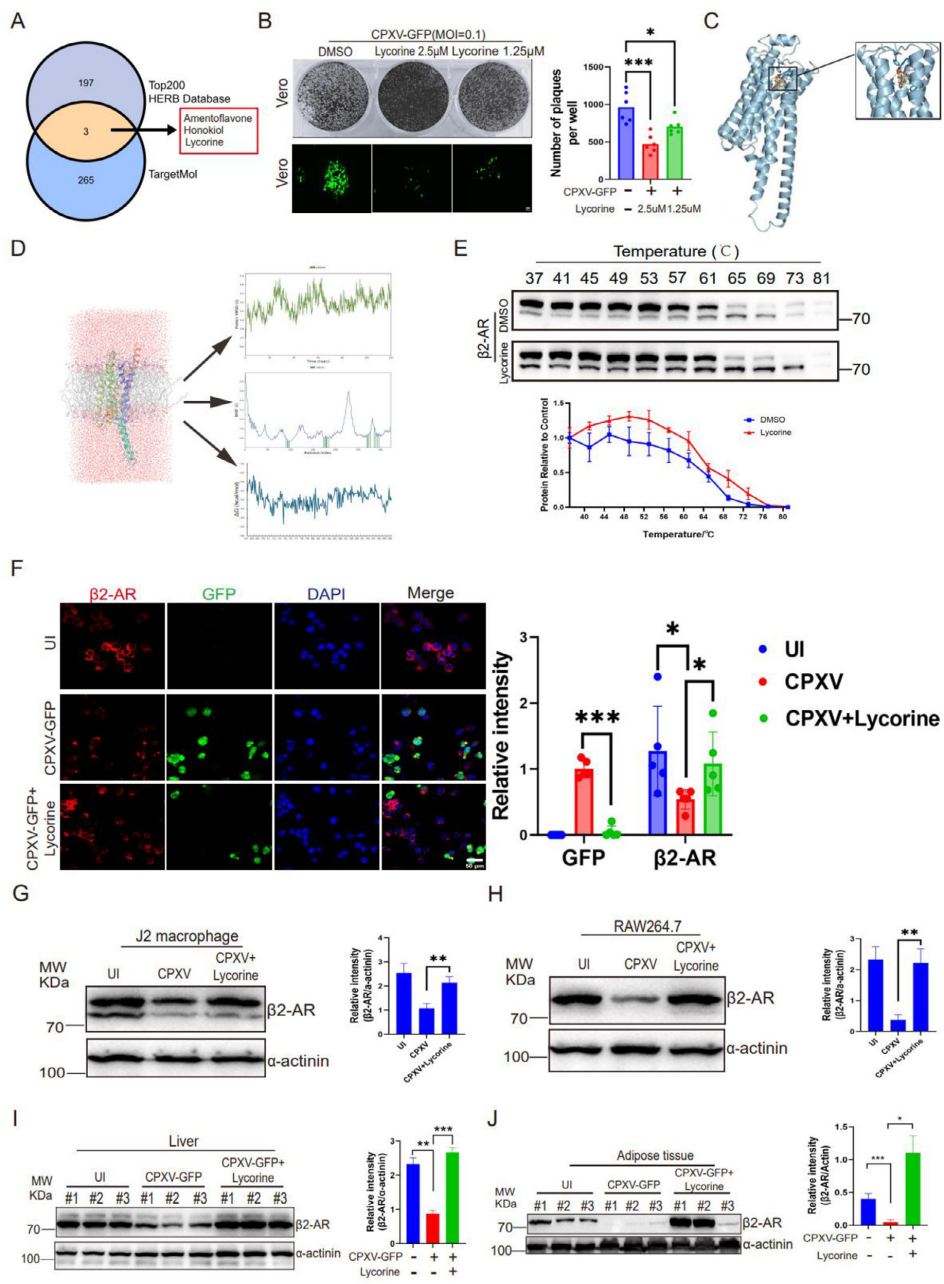
Identification of lycorine as a β2‐AR‐stabilizing antiviral compound against CPXV infection. (A) Screening of candidate small molecules with high binding affinity to β2‐AR via cross‐database analysis of the Herb and TargetMol compound libraries. (B) Antiviral activity validation of the candidate drug lycorine at different concentrations. (C) Binding conformation of lycorine within the β2‐AR_MOUSE protein structure. The overall β2‐AR protein is shown in light blue, with amino acid residues surrounding the ligand displayed in dark blue. The small molecule lycorine is represented with orange carbon (C), gray nitrogen (N), and red oxygen (O) atoms. Gray dashed lines indicate hydrophobic interactions, blue solid lines represent hydrogen bonds, green dashed lines denote *π–π* stacking, and yellow dashed lines represent salt‐bridge interactions. (D) Binding mode and dynamics analysis of lycorine with the β2‐AR dimer. (Left) Three‐dimensional structure of the protein‐ligand complex embedded in a cubic solvent model, with red points representing oxygen atoms of water molecules. (Top right) RMSD analysis of the protein backbone from molecular dynamics simulations. (Middle right) RMSF analysis of the protein backbone from molecular dynamics simulations. (Bottom right) Binding free energy analysis calculated by the MM/GBSA method. (E) Immunoblot analysis of the binding between lycorine and β2‐AR in Vero cells via CETSA. (F) Immunostaining assay was used to analyze the colocalization of β2‐AR (red) and GFP (green) with lycorine (2.5 µm) treatment. Scale bars, 50 µm. (G,H) Immunoblot analysis of the effect on lycorine treatment at the level of β2‐AR in J2 macrophages (G) and RAW264.7 (H). (I,J) Protein level of lycorine mediated modulation of β2‐AR expression in liver (I) and adipose tissue (J) in vivo (*n* =3/group). Data are presented as mean ± SEM. Individual data points represent biologically independent samples, and n represents the number of biologically independent animals as detailed above. Statistical analysis was determined using the unpaired two‐tailed *t*‐test. ^*^ *p* < 0.05, ^**^ *p* < 0.01, ^***^ *p* < 0.001, ^****^ *p* < 0.0001; *ns*, not significant.

Given the limitations associated with long‐term β2‐AR agonist therapy [37], we sought to identify alternative compounds capable of targeting β2‐AR while exerting antiviral activity. To this end, small‐molecule ligands associated with ADRB2 in the HERB database (https://herb.ac.cn) were retrieved and individually docked to the mouse β2‐AR (ADRB2) binding pocket using AutoDock Vina. Each compound was ranked according to its predicted binding affinity (binding energy), and the top 200 candidates with the most favorable docking scores were selected for further analysis. These prioritized compounds were then cross‐referenced with a library of 265 reported antiviral agents from the TargetMol database, yielding three overlapping candidates: Amentoflavone, Honokiol, and Lycorine (Figure 4A). The antiviral activities of these candidates were evaluated against CPXV infection in vitro. Neither Amentoflavone nor Honokiol exhibited appreciable antiviral activity at 10 µm (Figure S5C,D). In contrast, lycorine potently inhibited CPXV replication, with significant antiviral activity observed at concentrations as low as 1.25 µm (Figure 4B and Figure S5A). Cytotoxicity analysis revealed a CC_50_ of 20.15 µm and an EC_50_ of 1.07 µm (Figure S5B). These findings identified lycorine as the most promising β2‐AR targeting antiviral candidate.

To investigate the molecular basis of this activity, protein‐ligand interaction fingerprint (PLIF) analysis was performed to characterize interactions between lycorine and β2‐AR. Among the predicted binding poses, Asp113 and Phe193 emerged as the most frequently engaged residues, with lycorine forming a stable Arene‐H interaction with Phe193 (Figure 4C and Figure S5E,F). Molecular dynamics simulations further supported stable binding of lycorine within the β2‐AR binding pocket, particularly around Asp113 and Phe193, as demonstrated by RMSD, RMSF and interaction analyses (Figure 4D and Figure S5G).

To experimentally validate target engagement, we performed cellular thermal shift assays (CETSA) and drug affinity responsive target stability (DARTS) assays. Lycorine treatment significantly enhanced the thermal stability of β2‐AR and protected the receptor from proteolytic degradation (Figure 4E and Figure S5H). These complementary approaches provide cellular evidence that lycorine engages and stabilizes β2‐AR.

Collectively, these computational and biochemical analyses demonstrate that lycorine directly binds and stabilizes β2‐AR. Together with its potent antiviral activity, these findings identify lycorine as a promising β2‐AR targeting antiviral candidate for the treatment of CPXV infection.

### 2.5 Lycorine Exerts Post‐Entry Antiviral Activity Against Orthopoxvirus Infection

Having identified β2‐AR as a potential target associated with lycorine activity, we next investigated its functional effects during viral infection. In CPXV‐infected J2 and RAW264.7 macrophages, viral infection markedly reduced β2‐AR expression, whereas lycorine treatment effectively reversed this downregulation (Figure 4G,H). Immunofluorescence analysis further showed that lycorine restores the peripheral distribution of β2‐AR in infected cells (Figure 4F). Consistent with these in vitro findings, lycorine also restored β2‐AR expression in the liver and epididymal adipose tissues of CPXV‐infected mice (Figure 4I,J). Together, these findings indicate that lycorine stabilizes β2‐AR abundance under infected conditions.

**FIGURE 5:**
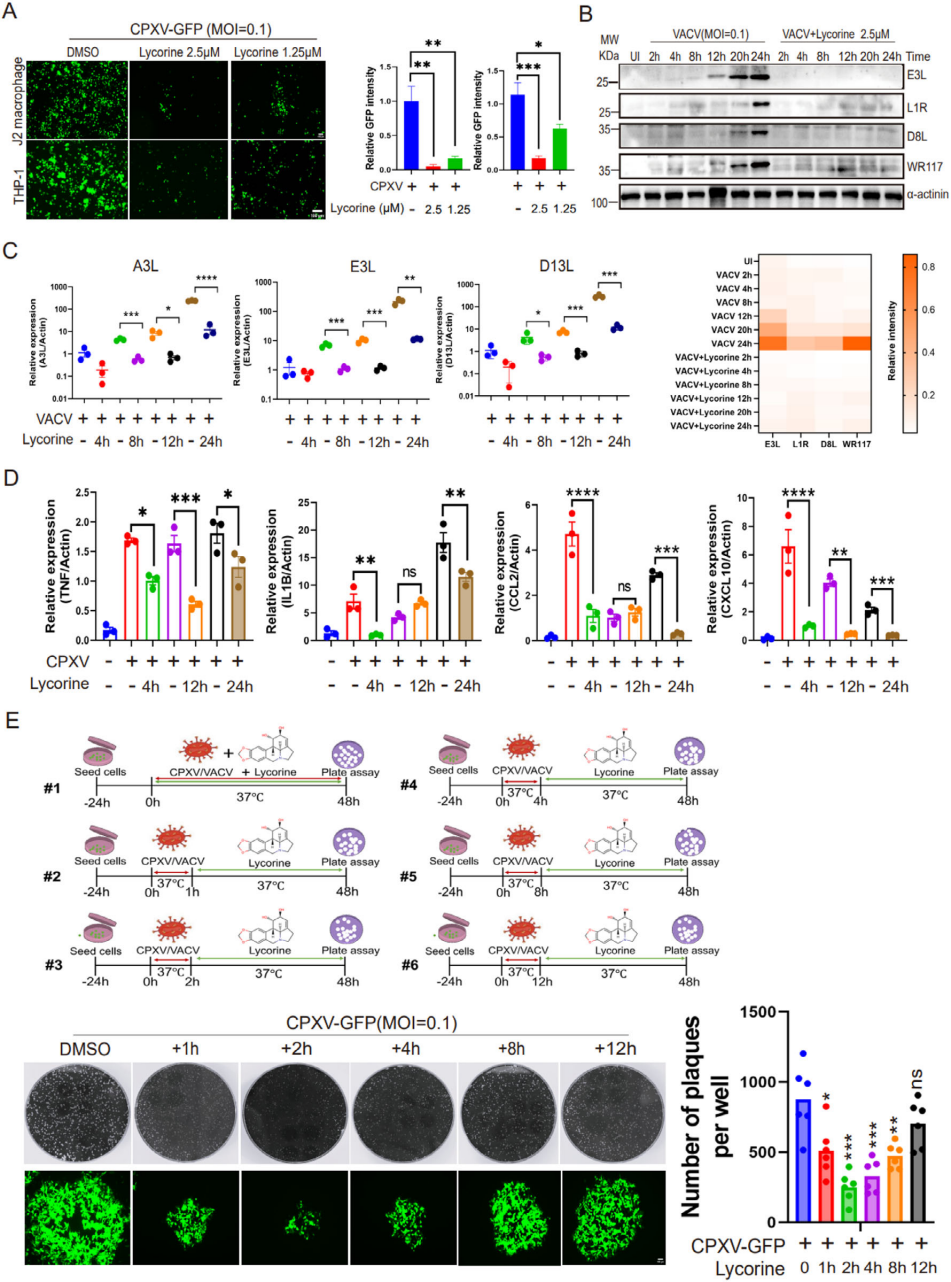
Lycorine exerts post‐entry antiviral activity against orthopoxvirus infection. (A) Potency of lycorine on CPXV‐induced viral replication in J2 macrophages and THP‐1 cells. (B) Analysis of viral protein expression in BS‐C‐1 cells post VACV infection over time. Viral proteins (E3L, L1R, D8L, WR117) were detected and quantified by immunoblot. (C) Time‐course analysis of viral gene expression in BS‐C‐1 cells after VACV infection. Viral genes (A3L, E3L, D13L) were detected and quantified by qRT‐PCR. (D) Effect of lycorine on the expression of inflammatory cytokines released after CPXV infection in J2 macrophages. (E) Assessment of the therapeutic effect of lycorine at different time points post‐CPXV infection. Viral replication was monitored by plaque assay (spots formation) and GFP fluorescence. Similar results were obtained from three biologically independent experiments (A–E). Data are presented as mean ± SEM. Individual data points represent biologically independent samples, and n represents the number of biologically independent animals as detailed above. Statistical analysis was determined using the unpaired two‐tailed *t*‐test. ^*^ *p* < 0.05, ^**^ *p* < 0.01, ^***^ *p* < 0.001, ^****^ *p* < 0.0001; *ns*, not significant.

We next evaluated the antiviral efficacy of lycorine against orthopoxvirus infection. Lycorine potently inhibited both CPXV and VACV replication, with significant antiviral activity observed at concentrations as low as 1.25 µm (Figure 4B and Figure S6A). Similar dose‐dependent antiviral effects were observed in THP‐1 cells and J2 macrophages, demonstrating broad activity across multiple cell types (Figure 5A). Consistent with these results, lycorine significantly reduced VACV protein expression and viral gene transcription in infected BS‐C‐1 cells (Figure 5B,C and Figure S6B). In addition, lycorine markedly attenuated CPXV‐induced inflammatory cytokine production (Figure 5D). These results suggest that lycorine exhibits potent antiviral activity while limiting excessive virus‐induced inflammatory responses during orthopoxvirus infection.

To define the stage of the viral life cycle targeted by lycorine, time‐addition experiments were performed. Lycorine retained substantial antiviral activity when administered up to 12 h post‐infection, whereas pretreatment before viral inoculation failed to confer protection (Figure 5E and Figure S6C). These findings suggest that lycorine does not interfere with viral attachment or entry but instead acts at post‐entry stage of infection. Together, these data indicate that lycorine suppresses intracellular viral replication and subsequent virus production.

Collectively, these findings demonstrate that lycorine preserves β2‐AR expression during infection and exerts potent post‐entry antiviral activity against orthopoxviruses.

By suppressing viral gene expression, viral protein synthesis, inflammatory responses, and post‐entry viral replication, lycorine emerges as a promising host‐directed therapeutic candidate for the treatment of poxvirus infection.

### 2.6 Lycorine Protects Against CPXV Infection Through Myeloid β2‐AR Signaling and CD8+ T Cell‐Dependent Immunity

**FIGURE 6:**
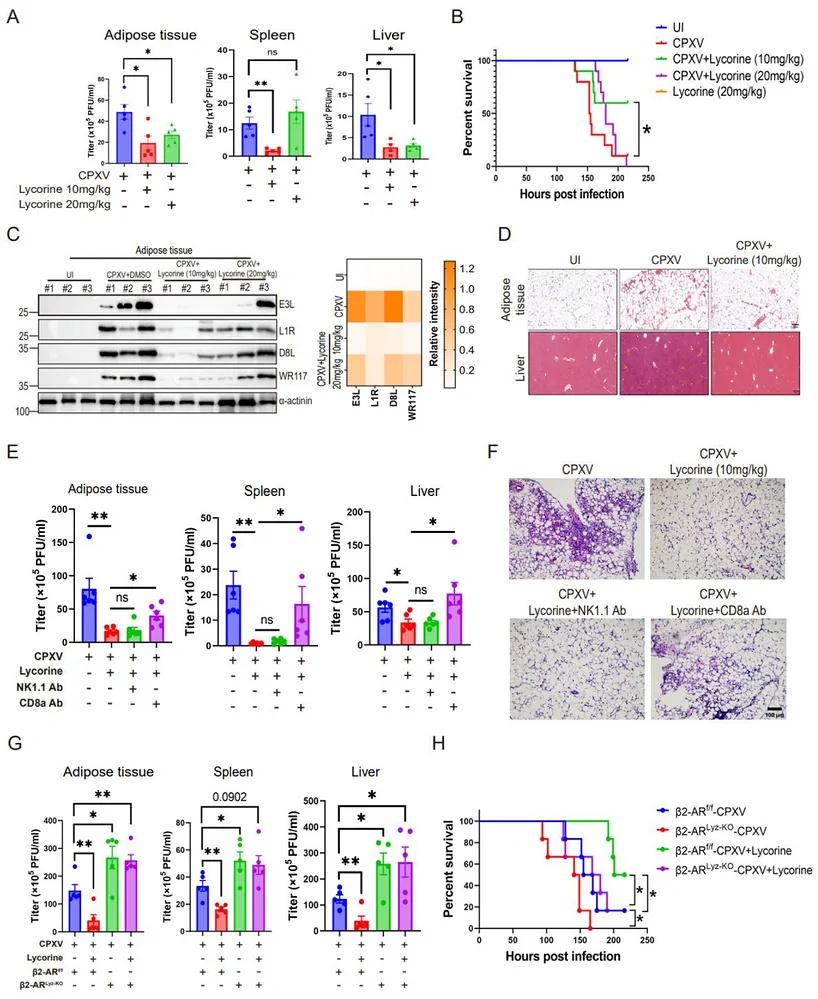
Lycorine protects against CPXV infection through myeloid β2‐AR signaling and CD8^+^ T cell‐dependent immunity. (A) Viral titers in the liver, spleen and adipose tissue of CPXV‐infected mice treated with lycorine (10 or 20 mg/kg) (*n* =5/group). (B) Survival of CPXV‐infected mice treated with lycorine treatment. Male mice were inoculated with CPXV and administered lycorine (10 or 20 mg/kg) or PBS (*n* =10/group). (C) Immunoblot analysis and quantification of viral protein (E3L, L1R, D8L, WR117) in epididymal adipose tissue. (D) Representative H&E staining of epididymal adipose tissue and liver from control, CPXV‐infected and lycorine‐treated (10 mg/kg) mice. Scale bars, 100 µm. (E) Viral titers in the liver, spleen and epididymal adipose tissue following CD8^+^ T cell or NK cell depletion in lycorine treated mice (10 mg/kg) (*n* =6 /group). (F) Representative H&E staining of epididymal adipose tissue from mice subjected to CD8^+^ T cell or NK cell depletion prior to CPXV infection. Scale bars, 100 µm. (G,H) Viral titers (G) and survival (H) of myeloid‐specific β2‐AR knockout (β2‐AR^Lyz‐KO^) and littermate control (β2‐AR^f/f^) mice following CPXV infection and lycorine treatment. *n* =5/group for viral titers and *n* =6/group for survival. Data are presented as mean ± SEM. n represents the number of biologically independent animals as detailed above. Statistical analyses were determined using unpaired two‐tailed Student's *t*‐test or log‐rank (Mantel‐Cox) test. ^*^ *p* < 0.05, ^**^ *p* < 0.01, ^***^ *p* < 0.001, ^****^ *p* < 0.0001; *ns*, not significant.

To evaluate the antiviral efficacy of lycorine in vivo, mice were treated with lycorine (10 or 20 mg/kg) beginning 5 days prior to CPXV challenge and continuing daily thereafter (Figure S7A,B). Lycorine at 10 mg/kg significantly reduced viral burden in the liver, spleen and epididymal adipose tissue and markedly improved survival following CPXV infection (Figure 6A,B). Consistently, viral proteins including L1R, D8L, E3L, and WR117 were substantially reduced in adipose tissues (Figure 6C), and similar reductions in these viral proteins were also observed in the liver (Figure S7C). In contrast, the 20 mg/kg dose increased viral burden and mortality, likely reflecting dose‐dependent toxicity. Although the underlying mechanism remains unclear, previous studies have reported neurotoxic effects associated with high dose lycorine administration [38]. Histological analysis further demonstrated that 10 mg/kg lycorine alleviated CPXV‐induced tissue damage, as evidenced by reduced inflammatory infiltration in epididymal adipose tissue and improved liver pathology (Figure 6D). Consistent with these findings, F4/80^+^ macrophage accumulation was markedly decreased in lycorine‐treated tissues (Figure S7D).

To identify the immune subsets responsible for lycorine‐mediated protection, CD8^+^ T cells or NK cells were depleted in lycorine‐treated, CPXV‐infected mice. Efficient depletion was confirmed by immunofluorescence staining (Figure S7E,F). Notably, CD8^+^ T cell depletion, but not NK cell depletion, largely abolished the antiviral effect of lycorine, resulting in elevated viral loads and exacerbated tissue inflammation (Figure 6E,F). These results indicate that CD8^+^ T cells are required for lycorine‐mediated antiviral protection, whereas NK cells play a minimal role.

Given the established role of β2‐AR signaling in antiviral immunity and CD8^+^ T cell responses [22], we next investigated whether β2‐AR is required for the antiviral activity of lycorine. Myeloid‐specific β2‐AR knockout mice were infected with CPXV and treated with lycorine. Deletion of myeloid β2‐AR markedly impaired the antiviral efficacy of lycorine, resulting in increased viral loads in the liver, spleen, adipose tissue, as well as loss of the survival benefit observed in littermate controls (Figure 6G,H). Together, these results demonstrate that myeloid β2‐AR is required for lycorine‐mediated protection against CPXV infection, with CD8^+^ T cells serving as essential contributors to its antiviral efficacy.

### 2.7 Lycorine Suppresses TBK1/IRF3‐Driven Inflammatory Signaling While Promoting β2‐AR‐Associated IFN/ISG Responses

**FIGURE 7:**
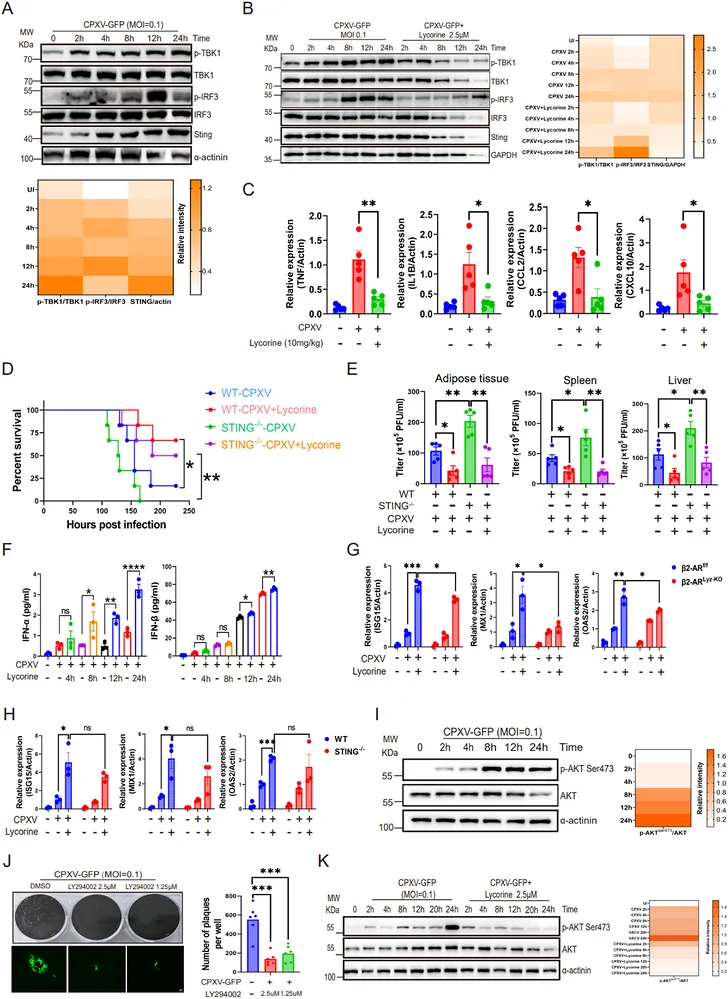
Lycorine suppresses TBK1/IRF3‐driven inflammatory signaling while promoting β2‐AR‐associated IFN/ISG responses. (A) Immunoblot analysis and quantification of STING, p‐TBK1, and p‐IRF3 in CPXV‐infected J2 macrophages (MOI=0.1) at the indicated time points. (B) Immunoblot analysis of STING, p‐TBK1 and p‐IRF3 in CPXV‐infected J2 macrophages treated with lycorine (2.5 µm). (C) qRT‐PCR analysis of inflammatory cytokine and chemokine expression in epididymal adipose tissue from CPXV‐infected mice with or without lycorine treatment (10 mg/kg). (D) Survival of WT and STING^−/−^ mice treated with vehicle or lycorine following CPXV infection (*n* = 6/group. Statistical significance was determined using the log‐rank (Mantel–Cox) test. (E) Viral titers in the liver, spleen, and epididymal adipose tissues of WT and STING^−/−^ mice treated with lycorine following CPXV infection (*n* = 5/group). (F) IFN‐α and IFN‐β levels in supernatants from CPXV‐infected J2 macrophages with or without lycorine treatment, measured by ELISA. (G) qRT‐PCR analysis of ISG15, MX1, and OAS2 expression in CPXV‐infected BMDMs from β2‐AR^f/f^ and β2‐AR^Lyz‐KO^ mice treated with lycorine for 24 h (*n* = 3/group). (H) qRT‐PCR analysis of ISG15, MX1, and OAS2 expression in CPXV‐infected BMDMs from WT and STING^−/−^ mice treated with lycorine for 24 h (*n* = 3/group). (I) Immunoblot analysis of AKT phosphorylation in CPXV‐infected cells (MOI=0.1) at the indicated time points. (J) Effects of the PI3K inhibitor LY294002 on CPXV infection, assessed by plaque assay and GFP fluorescence. (K) Immunoblot analysis and quantification of p‐AKT levels in CPXV‐ infected cells (MOI=0.1) treated with lycorine (2.5 µm) at the indicated time points. Data are presented as mean ± SEM. n represents the number of biologically independent animals as detailed above. Statistical analyses were determined using unpaired two‐tailed Student's *t*‐tests unless otherwise indicated. ^*^ *p* < 0.05, ^**^ *p* < 0.01, ^***^ *p* < 0.001, ^****^ *p* < 0.0001; *ns*, not significant.

To determine whether lycorine modulates innate antiviral immunity during poxvirus infection, J2 macrophages were infected with CPXV and treated with lycorine. CPXV infection induced progressive activation of the STING‐TBK1‐IRF3 axis, whereas lycorine markedly reduced TBK1/IRF3 phosphorylation (Figure 7A,B). Similar inhibitory effects were observed during VACV infection in BS‐C‐1 cells (Figure S8A,B). Consistent with the attenuation of virus‐induced innate inflammatory signaling, lycorine significantly reduced the expression of pro‐inflammatory cytokines and chemokines, including TNF‐α, IL‐1β, CCL2 and CXCL10 (Figure 7C). To determine whether STING contributes to the antiviral activity of lycorine, STING‐deficient mice were challenged with CPXV and treated with lycorine. Although STING deficiency increased viral susceptibility and mortality, lycorine retained its antiviral efficacy, significantly improving survival and reducing viral loads in multiple tissues (Figure 7D,E). These findings indicate that STING is not essential for the antiviral efficacy of lycorine.

We next examined type I IFN responses during CPXV infection. Lycorine treatment significantly increased IFN‐α and IFN‐β production in CPXV‐infected macrophages, with sustained elevation throughout infection (Figure 7F). Lycorine also enhanced the expression of the antiviral ISGs, including ISG15, MX1 and OAS2 (Figure 7G,H). To determine whether this response involves β2‐AR signaling, BMDMs from myeloid‐specific β2‐AR knockout mice were infected with CPXV and treated with either salmeterol or lycorine. Both compounds robustly induced ISG15, MX1 and OAS2 expression in control BMDMs, whereas this induction was markedly attenuated in β2‐AR‐deficient cells (Figure 7G and Figure S8C). However, residual induction, particularly of ISG15, remained detectable, indicating that β2‐AR contributes to, but does not fully account for, lycorine‐mediated ISG induction. We next examined whether STING is required for this response. Neither salmeterol‐ nor lycorine‐induced ISG expression was substantially impaired in STING‐deficient macrophages (Figure 7H and Figure S8D), although a modest reduction in the induction of some ISGs was observed. Together, these results indicate that β2‐AR signaling contributes to lycorine‐enhanced IFN/ISG responses, whereas STING is largely dispensable. The persistence of ISG induction in both β2‐AR‐ and STING‐deficient macrophages further suggests that lycorine may engage additional regulatory mechanisms or parallel antiviral signaling pathways that remain to be defined.

Given the reported involvement of β2‐AR‐associated signaling in PI3K‐AKT regulation, we next examined whether AKT signaling is altered during poxvirus infection and lycorine treatment. CPXV and VACV infection induced robust AKT phosphorylation in a time‐ and virulence‐ dependent manner (Figure 7I and Figure S8E–G) and pharmacological inhibition of PI3K or AKT reduced viral replication (Figure 7J and Figure S8H), indicating that AKT activation contributes to poxvirus infection. Notably, lycorine treatment significantly attenuated virus‐induced AKT phosphorylation (Figure 7K and Figure S8I,J). These findings suggest that lycorine suppresses pro‐viral AKT signaling during poxvirus infection, although whether this effect is directly mediated through β2‐AR stabilization requires further investigation.

Collectively, these findings indicate that lycorine exerts potent antiviral and immunomodulatory activities during poxvirus infection. Lycorine attenuates virus‐induced inflammatory signaling while enhancing antiviral IFN/ISG responses through β2‐AR‐associated and additional regulatory mechanisms, and suppresses viral‐associated PI3K‐AKT activation. Given the persistence of IFN/ISG induction in β2‐AR‐ and STING‐deficient macrophages, together with the simultaneous suppression of STING‐TBK1‐IRF3 signaling, these observations suggest that lycorine likely regulates antiviral immunity through multiple molecular targets or parallel signaling pathways, which warrant further investigation.

## 3 Discussion

The recent resurgence of orthopoxviruses, exemplified by the global mpox outbreak, has underscored their significant public health threat [39]. These viruses cause substantial economic burdens through healthcare and containment costs and severely strain pandemic preparedness systems. Compounding this challenge is a limited clinical arsenal. While drugs like Tecovirimat provide a foundational therapy, their utility is constrained by emerging resistance and dose‐limiting toxicities, such as the nephrotoxicity associated with Cidofovir [40]. This therapeutic gap highlights the urgent need for novel, safer, and more effective antiviral strategies, particularly those employing host‐directed mechanisms which may be less susceptible to viral mutation.

Inspired by the emerging paradigm of targeting host proteins with small molecule stabilizers, a strategy exemplified by compounds that stabilize RIG‐I oligomerization [41], YAP protein [42], or SKIC8 [43], we identify lycorine as a promising host‐targeting antiviral compound that effectively inhibits orthopoxviral infection, a natural alkaloid with known broad spectrum antiviral activity, as a high‐affinity candidate [27, 44, 45, 46, 47]. A notable finding is that lycorine interacts with β2‐AR and restores its plasma membrane localization and protein abundance during CPXV infection. Although the precise structure basis of this interaction remains to be elucidated, our findings suggest that lycorine functions as a β2‐AR stabilizer rather than a conventional agonist. β2‐AR abundance is dynamically regulated by receptor internalization, ubiquitination and lysosomal degradation following cellular stress or sustained stimulation [48]. Consistent with this regulatory paradigm, CPXV infection markedly reduced β2‐AR protein levels, whereas lycorine effectively restored receptor expression both in vitro and in vivo. Those observations support a model in which lycorine binding stabilizes β2‐AR in a conformation that is less susceptible to virus‐induced internalization and degradation, thereby preserving cell‐surface receptor availability and enhancing β2‐AR‐associated antiviral responses. The precise molecular mechanism underlying β2‐AR stabilization remains to be determined. Lycorine may allosterically stabilize the receptor [49], reduce β‐arrestin‐dependent receptor internalization [50], interfere with ubiquitin‐mediated degradation or alter endolysosomal trafficking and autophagic turnover [51]. Distinguishing among these possibilities will require future structural, biophysical and receptor trafficking studies. Nevertheless, our findings establish β2‐AR as a previously unrecognized molecular target of lycorine and provide a mechanistic framework for exploiting receptor stabilization as a host‐directed antiviral strategy against orthopoxvirus infection.

Mechanistically, our study suggests that lycorine remodels host antiviral responses through multiple functionally distinct pathways. Lycorine restored β2‐AR expression in CPXV‐infected macrophages, while simultaneously attenuating virus‐induced AKT activation, suppressing STING‐TBK1‐IRF3‐associated inflammatory signaling, and enhancing type I IFN production together with antiviral ISG expression. Although β2‐AR stabilization is required for the antiviral activity of lycorine, whether the regulation of AKT and inflammatory signaling represents a direct consequence of β2‐AR restoration or an independent action of lycorine remains to be determined. These findings suggest that lycorine appears to simultaneously restrain excessive inflammatory responses while preserving antiviral immunity, thereby promoting a more balanced host response during infection.

An unexpected finding was that lycorine enhanced IFN/ISG responses despite suppressing virus‐induced activation of the STING‐TBK1‐IRF3 pathway. This observation suggests that the antiviral activity of lycorine cannot be explained solely by modulation of the canonical STING‐TBK1‐IRF3 signaling cascade. Instead, lycorine likely exerts pleiotropic immunomodulatory effects by coordinating multiple signaling pathways. Consistent with this interpretation, residual ISG induction remained detected in both β2‐AR‐ and STING‐deficient macrophages, indicating that although β2‐AR contributes to lycorine‐mediated antiviral responses, additional signaling pathways or molecular targets are also involved. Furthermore, because lycorine markedly suppresses viral replication, the reduced phosphorylation of TBK1 and IRF3 observed at later stages of infection may, at least in part, reflect diminished viral burden and consequently reduced activation of upstream innate immune sensors.

The mechanisms by which lycorine enhances IFN and ISG expression independently of the canonical STING pathway remain to be elucidated. β2‐AR signaling is known to activate both the Gαs‐PKA‐CREB and β‐arrestin‐dependent MAPK pathways [52, 53]. While IRF1 has been shown to drive ISG expression independently of IRF3 or IRF7 [54]. Whether these pathways contribute to the antiviral effects of lycorine warrants further investigation. Moreover, given the well‐documented cell‐type‐ and context‐dependent nature of β2‐AR signaling, the relative contribution of this receptor on distinct immune subsets beyond macrophages, including CD8^+^ T cells and dendritic cells should also be explored. Elucidating these additional molecular mechanisms will further clarify how lycorine coordinates antiviral immunity and inflammatory responses during poxvirus infection.

Our findings also provide mechanistic insight into the long‐recognized association between psychological stress and increased susceptibility to viral infection. Although stress hormones such glucocorticoids and catecholamines are known to modulate antiviral immunity [18, 19], the molecular basis for this effect has remained incompletely understood. We demonstrate that chronic psychological stress promotes CPXV infection through autophagy‐dependent degradation of β2‐AR, thereby identifying β2‐AR as a critical molecular link connecting neuroendocrine signaling with innate antiviral defense. These findings further suggest that orthopoxviruses exploit this stress responsive pathway by actively inducing β2‐AR degradation to facilitate infection. Although our data indicates that this process may involve a viral‐protease‐dependent mechanism, the identity of the responsible viral factor remains unknown and represents an important direction for future investigation.

Despite the potent antiviral activity of lycorine, our in vivo studies revealed dose‐dependent toxicity at doses exceeding 10 mg/kg, highlighting the need to improve its therapeutic window. Future medicinal chemistry efforts should therefore focus on optimizing lycorine derivatives that retain β2‐AR‐stabilizing activity while minimizing toxicity. Collectively, our study identifies β2‐AR as a previously unrecognized host factor targeted by orthopoxviruses and highlights pharmacological stabilization of β2‐AR as a potential host‐targeted antiviral strategy against orthopoxvirus infection.

## 4 Conclusions

**FIGURE 8:**
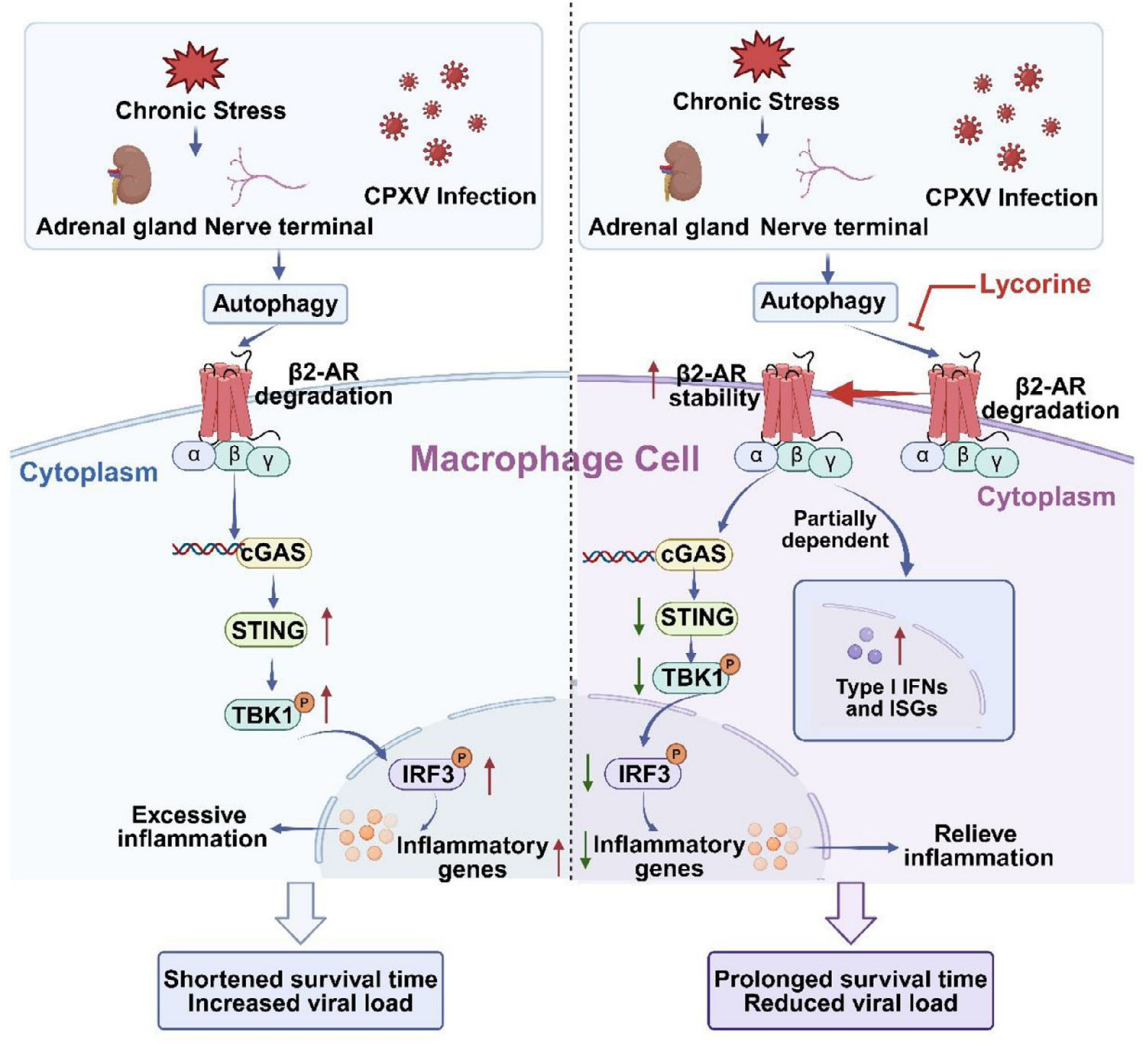
Lycorine stabilizes β2‐AR to protect against poxvirus infection. Chronic stress exacerbates poxvirus infection by promoting autophagy‐mediated degradation of the β2‐adrenergic receptor (β2‐AR), leading to impaired antiviral defense, enhanced viral replication, excessive inflammatory activation and increased mortality. Pharmacological stabilization of β2‐AR by the natural compound lycorine preserves β2‐AR availability, restricts viral replication, modulates host immune responses, attenuates excessive inflammation and improves survival following poxvirus infection.

Our study identifies a neuroimmune regulatory axis linking chronic stress, β2‐AR downregulation, and poxvirus pathogenesis. We further demonstrate that lycorine functions as a β2‐AR‐stabilizing compound with potent antiviral activity against orthopoxvirus infection. By preserving β2‐AR availability, restricting viral replication, and modulating host immune signaling, lycorine enhances antiviral IFN/ISG responses through β2‐AR‐associated and additional regulatory mechanisms while restraining excessive inflammatory activation. These findings provide new insights into stress‐associated susceptibility to viral infection and highlight β2‐AR stabilization as a potential host‐directed therapeutic strategy for orthopoxvirus infection and other stress‐associated viral diseases (Figure 8).

## 5 Materials and Methods

### 5.1 Cell Culture

J2 cells were immortalized by retroviral infection; RAW264.7 and THP‐1 cells, which are highly vulnerable to CPXV infection, were used. J2 macrophages and RAW264.7 were maintained in Dulbecco's Modified Eagle Medium(DMEM, Pricella, Wuhan, CN) supplemented with 10% heated‐inactivated fetal bovine serum (FBS). The human monocytic cell line THP‐1 was cultured in Roswell Park Memorial Institute 1640 Medium(RPMI 1640, Pricella, Wuhan, CN)supplemented with 10% FBS. Both J2 macrophages and RAW264.7 cells, as well as THP‐1 cells, are known to be highly susceptible to CPXV infection. All cell lines were incubated at 37°C in a humidified 5% CO_2_ incubator. For virus propagation and titration, Vero (a monkey kidney cell line) and BS‐C‐1 cell were employed. Vero cells were cultured in DMDM, while BS‐C‐1 cells were maintained in Minimum Essential Medium (MEM, Pricella, Wuhan, CN), both supplemented with 10% heated‐inactivated FBS. Vero cells were used for viral titer determination, whereas BS‐C‐1 cells were utilized for both viral amplification and titer determination.

### 5.2 Virus Strain

CPXV (Bright Red Strain), CPXV‐GFP, VACV (Western Reserve Strain) have been previously described by Mohamed et al. [55]. The viruses were amplified and passaged in BS‐C‐1 cells at a multiplicity of infection (MOI) of 10. After 48 h, the heavily infected BS‐C‐1 cells were scraped into centrifuge tubes and centrifuged at 1,500 rpm for 5 min. The resulting pellet was resuspended in MEM supplemented with 2.5% FBS. The suspension was then subjected to three freeze‐thaw cycles.

For viral infection and drug screening experiments, all compounds were first determined to be non‐cytotoxic at a concentration of 2.5 µm; this concentration was therefore used in subsequent viral inhibition assays. Vero cells were infected with CPXV at an MOI of 0.1, and BS‐C‐1 cells were also infected with VACV (MOI═0.1). After 2 h, the supernatant was removed and replaced with medium containing 2.5% serum and each respective small molecule drug. Following 48 h of incubation, the cells were fixed with 4% paraformaldehyde for 20 min and then stained with crystal violet. Plaques were counted visually, and the viral inhibition rate was calculated accordingly.

### 5.3 Antibodies and Reagents

Monoclonal antibody (mAb) against VACV proteins E3L (CSB‐PA322729ZA01VAA), L1R (CSB‐PA324949ZA01VAA), VACWR117(CSB‐PA356237XA01VAA) and D8L (CSB‐PA322653LA01VAA) were obtained from CUSABIO, China. p‐TBK1 (5483s), TBK1 (3504s), IRF3 (4302s) and STING (13647s) were obtained from CST, USA. p‐IRF3(# ET1608‐22) was purchased from HUABIO, China. p‐AKT (F1644) and AKT (F0670) were obtained from Selleck, USA. β2‐AR antibody (BS1716) was obtained from Bioworld, USA. α‐actin antibody (23660‐1‐AP) was purchased from Proteintech, USA. Lycorine(#T3324) and β2‐AR agonists, including salmeterol (43229‐80‐[7]) and formoterol hemifumarate ([18559]‐94‐9), were all obtained from TargetMol; LY294002 (HY‐10108), BafA1 (HY‐100558), CQ (HY‐17589A), MG132 (HY‐13259) were purchased from MedChemExpress (Shanghai, CN).

### 5.4 Western Blot Analysis

Tissue and cell samples were lysed on ice for 3 min using SDS lysis buffer containing protease and phosphatase inhibitors (composition: 10% SDS, pH 7.4 Tris‐HCl, ddH_2_O), followed by centrifugation at 12 000 rpm for 5 min. The protein supernatant was collected, and its concentration was determined using a BCA protein assay kit (Thermo Fisher, Cat# 23225, USA). Proteins were separated by SDS‐PAGE and transferred to an NC membrane (Millipore, Cat# HATF00010, USA). The membrane was blocked with 5% skim milk at room temperature for 2 h, then incubated with a specific primary antibody overnight at 4°C with gentle shaking. After washing three times with TBST, the membrane was incubated with a secondary antibody at room temperature for 1 h to amplify the signal. The antibodies utilized for western blot in this study are as follows: E3L (from CUSABIO, CSB‐PA322729ZA01VAA); D8L (from CUSABIUO, CSB‐PA322653LA01VAA); L1R (from CUSABIO, CSB‐PA324949ZA01VAA); WR117 (from CUSABIO, CSB‐PA356237XA01VAI); β2‐AR (from Bioworld, BS1716); p‐TBK1 (from CST,5483s); TBK1 (from CST,3504s); p‐IRF3 (from CST,4947s); IRF3 (from CST,4302s); STING (from CST,13647s); p‐AKT (from Selleck, F1644); AKT (from Selleck, F0670);α‐actinin (from Proteintech, 23660‐1‐AP). Chemiluminescence signals were detected by means of the Immobilon ECL Ultra Western HRP Substrate from Millipore.

### 5.5 Preparation of Primary Cells

Mice were anesthetized via inhalation anesthesia and euthanized by cervical dislocation. Following fixation in a supine position, the lower abdominal and hind limb regions were disinfected with 75% ethanol. The skin over the hind limbs was incised, and muscle tissue surrounding the femur and tibia was meticulously removed under strict aseptic conditions. Care was taken to avoid bone fracture and marrow leakage to prevent contamination. Bones were surface sterilized with 75% ethanol and transferred to ice‐cold RPMI 1640 medium. After excision of both epiphyses, marrow was flushed with ice‐cold RPMI 1640 medium using a syringe, repeated 2–3 times until bone blanching. The harvested marrow was gently dissociated by pipetting with a 1 mL tip and filtered through a 70 µm cell strainer. Cells were pelleted by centrifugation at 1500 rpm for 5 min, resuspended in RPMI 1640 medium supplemented with 20% L929 liquid supernatant recombinant GM‐CSF, and plated in culture dishes. Cultures were maintained at 37°C under 5% CO_2_, with medium replenishment every three days. Bone marrow‐derived macrophages (BMDMs) were obtained after 14 days of differentiation.

### 5.6 20% L929 Liquid Supernatant Preparation

The L929 cell line was first resuscitated and cultured. Upon achieving adequate density, the cells were passaged at a 1:3 ratio into 10‐cm dishes. By the second passage, all dishes had reached full density. One dish was used for continued passaging, while the other two were maintained with fresh medium for an additional 5 days. The conditioned medium was then collected, centrifuged at 1500 rpm for 5 min, and the supernatant was diluted into RPMI 1640 medium supplemented with 10% FBS and 1% penicillin‐streptomycin (PS). This supplemented medium was subsequently used for the culture of BMDMs. It should be noted that the functional state of L929 cells critically influences both the adherence efficiency and kinetics of BMDMs.

### 5.7 Mouse Infection and Viral Titer Determination

C57BL/6J mice were purchased from the Experimental Animal Center of the College of Medicine, Xi'an Jiaotong University (Xi'an, China). Mice were housed under controlled conditions at a constant temperature of 25°C with a 12 h artificial light/dark cycle, where the light period commenced at 8:00 AM daily. Meanwhile, for generating macrophage‐specific β2‐AR knockout mice, β2‐AR^fl/fl^ mice were crossed with Lyz2‐iCre^+/+^ mice. The resulting β2‐AR^fl/+^Lyz2‐iCre^+/−^ offspring were backcrossed with β2‐AR^fl/fl^ mice. After genotyping, mice with the genotypes β2‐AR^fl/fl^Lyz2‐iCre^+/+^ (β2‐AR^Lyz‐KO^) and β2‐AR^fl/fl^Lyz2‐iCre^−/−^ were identified as the experimental and control groups, respectively, and used for viral infection experiments.

Age‐matched mice were randomly assigned to different experimental groups. At 8 weeks of age, mice in each group were exposed to distinct stress environments, after which each mouse was intraperitoneally injected with 100 µL of CPXV containing 1×10^7^ PFU/mL. Liver, spleen, and epididymal adipose tissues were harvested at corresponding time points for viral titer determination in mouse organs. The tissue samples were placed in DMEM supplemented with 2.5% fetal bovine serum (FBS) and 5% penicillin‐streptomycin (PS), homogenized with beads at 4°C, and subsequently centrifuged at 2000 rpm for 20 min. The viral supernatant was retained, serially diluted, and added to Vero cells that had been seeded the previous day at a density of 5×10^5^ cells/mL. After 2 days of infection, the cells were fixed with 4% paraformaldehyde (PFA) for 20 min and stained with 0.1% crystal violet for 3 min, followed by rinsing under running water to visualize and quantify plaque‐forming units (PFU). Meanwhile, survival analyses were conducted under identical experimental conditions.

When studying the effects of lycorine on poxvirus infection, a prophylactic intraperitoneal administration of lycorine (10 and 20 mg/kg) was initiated 5 days in advance. On day 5, CPXV infection was performed, followed by continuous lycorine treatment until organ collection at 3.5 days for viral titer assays and until the survival study was concluded.

### 5.8 Behavioral Experiments

In this study, forty 8‐week‐old male C57BL/6J mice were randomly divided into a foot shock stress (FS) group and an enriched environment (EE) group, with 20 mice per group. FS mice received a daily foot shock stimulus (0.5 mA, 10 s) for 7 consecutive days, while EE mice were housed in an enriched environment equipped with running wheels, toys, and tunnels for 5 weeks. Anxiety‐like behavior was assessed using the open field test: mice were placed in the center of a 40 cm × 40 cm × 40 cm arena, and total distance moved, time spent in the central zone, and number of entries into the central zone were recorded during a 10 min session. The elevated plus maze was used to further validate anxiety behavior: mice were placed in the central area of a maze elevated 50 cm above the floor (with two open arms and two enclosed arms), and time spent in open arms, number of open arm entries, and open arm time ratio were recorded for 10 min.

### 5.9 Quantitative PCR

Total RNA was extracted from 20 mg of fresh tissue or cell samples using TRIZOL reagent (ABclonal, Wuhan, China), and all subsequent procedures were strictly performed according to the manufacturer's instructions. RNA concentration and purity were detected using a Nanodrop system. The RNA was reverse‐transcribed into cDNA using ABScript III RT Master Mix for qPCR, and amplification was conducted with 2X Universal SYBR Green Fast qPCR Mix (ABclonal) on a Bio‐Rad CFX Connect Real‐Time PCR Detection System. The expression levels of cytokines, chemokines, virus‐encoded genes, and ADRB2 were normalized to the reference gene β‐actin. The primer sequences used in this study are provided in the Key Resources Table.

### 5.10 Histology and Immunofluorescence

Freshly isolated mouse epididymal adipose tissue was fixed in 4% paraformaldehyde, embedded in paraffin, and sectioned for hematoxylin and eosin (H&E) staining. For immunofluorescence, paraffin sections were subjected to deparaffinization, rehydration, and antigen retrieval, followed by blocking with 5% BSA for 1 h. The sections were then incubated overnight at 4°C with a primary antibody against F4/80 (1:500, No. 28463‐1‐AP, Proteintech, China). After rewarming the sections for 30 min, a diluted Cy3‐conjugated secondary antibody was applied and incubated at 37°C for 1 h. Following washes, diluted DAPI was added for nuclear staining, and the slides were mounted. Finally, the samples were observed under a fluorescence microscope (Thermo Fisher, USA).

For cellular immunofluorescence, J2 and RAW264.7 cells were seeded at a density of 2 × 10^4^ cells/mL one day in advance. After CPXV infection and lycorine treatment, the cells were washed with cold PBS, fixed with 4% PFA for 15 min, permeabilized with 0.25% Triton X‐100 for 20 min, and blocked with 5% BSA for 30 min. Subsequently, the cells were incubated overnight at 4°C with a primary antibody against β2‐AR (1:200, BS1716, Bioworld, USA).

### 5.11 Cell Viability and Virus Inhibition Assays

Vero cells were seeded into 96‑well plates at a density of 1 × 10^4^ cells per well and cultured overnight at 37°C in a 5% CO_2_ incubator. After cell attachment, the medium was replaced with fresh medium containing serial twofold dilutions of lycorine (concentration range: 0.1–100 µm). Untreated cells and medium‑only wells were included as controls. Following 48 h of incubation, CCK‑8 solution (10 µL) was added to each well, and the plates were incubated for an additional 2 h at 37°C. Absorbance was measured at 450 nm using a microplate reader. Cell viability was calculated as: (OD experimental—OD blank) / (OD control—OD blank) × 100%. The 50% cytotoxic concentration (CC_50_) was derived from the dose‑response curve by nonlinear regression analysis.

For antiviral activity assessment, Vero cells were seeded and cultured as described above. After infection with CPXV at a multiplicity of infection (MOI) of 0.1, serial dilutions of lycorine were added. Following 2 h of virus adsorption, the medium was replaced with maintenance medium containing corresponding drug concentrations, and the cells were cultured for another 48 h. Viral replication was quantified by plaque reduction assay or fluorescence‑based detection (for recombinant virus). Virus‑infected cells without drug treatment served as positive controls, and mock‑infected cells were used as negative controls. The 50% effective concentration (EC_50_) was calculated from the inhibition curve. All experiments were performed in triplicate, and data were expressed as mean ± standard deviation.

### 5.12 Receptor Protein Preparation

The amino acid sequence of the mouse β_2_‐adrenergic receptor (ADRB2_MOUSE) was retrieved from the UniProt database (https://www.uniprot.org/). A homology comparison using the SWISS‐MODEL platform indicated that the protein shared 84.06% sequence similarity with the human ADRB2 crystal structure (PDB ID: 3PDS). The 3PDS structure contains an endogenous ligand (represented as green spheres), suggesting that this region corresponds to a conserved ligand‐binding pocket. Subsequently, the AlphaFold‐predicted structure of ADRB2_MOUSE was downloaded from the UniProt database. The potential binding sites on the protein surface were analyzed using the SiteFinder module in MOE 2022 (Chemical Computing Group, Montreal, Canada). Two promising binding pockets were identified as potential sites for inhibitor screening. Among them, Site 1 was located adjacent to the endogenous ligand‐binding region in the 3PDS structure and extended toward the extracellular domain, indicating that this site may serve as a key pocket suitable for small‐molecule inhibitor binding.

Before molecular docking, the ADRB2_MOUSE structure was preprocessed using the QuickPrep module in MOE, including bond order correction, hydrogen addition, disulfide bond assignment, hydrogen‐bond network optimization, and energy minimization, to ensure structural stability and proper protonation under physiological conditions.

### 5.13 Ligand Preparation

Small‐molecule ligands targeting the ADRB2 protein were obtained from the HERB database (https://herb.ac.cn, accessed on November 6, 2025). The 3D structures and canonical SMILES of these compounds were verified and standardized using the PubChem database to ensure structural accuracy and correct stereochemistry. Subsequently, Open Babel 3.1.1 was used to preprocess the ligands by adding hydrogen atoms and converting file formats to generate input files compatible with molecular docking.

### 5.14 Molecular Docking

Molecular docking simulations were performed using AutoDock Vina 1.2.7. A cubic grid box with dimensions of 20 × 20 × 20 Å^3^ was defined around the Site 1 binding pocket, and the exhaustiveness parameter was set to 8 to ensure sufficient conformational sampling.

### 5.15 ELISA

Mouse sera were assayed by ELISA kit (Elabscience, Wuhan, CN) for Norepinephrine and Epinephrine according to the manufacturer's instructions using a Gen5 plate reader.

### 5.16 Protein Thermal Shift Assay

Vero cells were plated in 10 cm dish at a density of 1 × 10^7^ cells per dish and were incubated overnight, followed by treatment with either 10 µm lycorine or an equal volume of DMSO control for 24 h under standard conditions (37°C, 5% CO_2_). After treatment, the cells were washed twice with pre‐chilled PBS, harvested via PBS suspension, and centrifuged at 3000 rpm for 5 min. The pellet was resuspended in PBS containing protease inhibitor and divided into 12 aliquots for thermal challenge across a gradient of temperatures (37°C‒81°C in 4°C increments). Each aliquot was heated for 3 min at the target temperature, immediately cooled on ice for 3 min, and subjected to three freeze‐thaw cycles in liquid nitrogen to ensure lysis. Lysates were then centrifuged at 20 000 g for 20 min at 4°C to separate soluble proteins, and the resulting supernatant was mixed with loading buffer for downstream protein analysis.

### 5.17 Drug Affinity Responsive Target Stability

Vero cells were seeded in 10 cm culture dishes at a density of 1 × 10^7^ cells per dish and incubated overnight under standard conditions (37°C, 5% CO_2_). Following three times with ice‐cold PBS, cells were lysed in M‐PER extraction reagent supplemented with protease inhibitors (500 µL per dish) by repeated aspiration through an insulin syringe. The lysates were centrifuged at high speed, and the supernatant was collected for protein quantification. Protein concentrations were normalized to 5 µg/µL.

Cell lysates were treated with 10 µm lycorine or an equal volume of DMSO for 2 h at 37°C. Proteinase was serially diluted at ratios of 1:100, 1:200, 1:500, and 1:1000. Aliquots of 50 µL lysate were mixed with corresponding dilutions of protease and reacted at room temperature for 30 min. The digestion was terminated by adding 20× protease inhibitors. After subsequent centrifugation, the supernatant was combined with 5× loading buffer, denatured at 100°C for 10 min, and prepared for subsequent protein analysis.

### 5.18 Virus Inactivation With 0.1% BPL

A 0.1% β‑propiolactone (BPL) solution was pre‑cooled on ice and then added dropwise to the CPXV stock solution with gentle stirring to achieve a final BPL concentration of 0.1%. The mixture was incubated at 4°C for 24 h with continuous agitation to ensure complete viral inactivation. After inactivation, the mixture was transferred to a 37°C water bath and shaken for 2 h to hydrolyze residual BPL. Inactivation efficiency was confirmed by inoculating the treated sample onto Vero cell monolayers and observing the absence of cytopathic effect (CPE).

### 5.19 Statistical Analysis

Statistical analysis was performed using GraphPad Prism 8.0. Data are presented as mean ± SEM. Comparisons between two groups were made using unpaired two‐tailed Student's *t*‐tests with a 95% confidence interval. The variable “n” refers to the number of animals per experimental group. Detailed statistical parameters are provided in the corresponding figure legends. A *p*‐value less than 0.05 was considered statistically significant (^*^ *p* < 0.05, ^**^ *p* < 0.01, ^***^ *p* < 0.001, ^****^ *p* < 0.0001). Survival analysis was conducted using the Kaplan–Meier method, and the curve was generated with GraphPad Prism 8.0.

## Author Contributions

**Zhijun Liu**, **Yingli He** and **Rui Yang** conceptualized and designed the project. Yingli He contributed to manuscript review and editing. **Xin Zhang**, **Shuqin Xu**, and **Zixuan Zhang** performed the experiments, analyzed the data, and drafted the initial manuscript. **Xiaoqin Wang** and **Tianxiao Huang** assisted with experimental procedures and data analysis. **Peihua Wang Yijia Yuan**, and **Chenwen Wan** were responsible for histological section evaluation. **Huini Yang** and **Faiza Atique** contributed to sample collection and data organization. All authors made substantial contributions to the work and approved the final version of the manuscript.

## Ethics Statement

This study underwent review and received approval from the First Affiliated Hospital of Xi'an Jiaotong University (approval number: XJTUAE2024619).

## Conflicts of Interest

The authors declare no conflicts of interest.

## Supporting information




**Supporting File**: advs77306‐sup‐0001‐SuppMat.docx.

## Data Availability

The data that support the findings of this study are available from the corresponding author upon reasonable request.
