# Supplementary material for: Targeting β2‐Adrenergic Receptor Stability With Lycorine Reveals a Host‐Directed Pathway for Antiviral Defense Against Poxviruses
Source: Adv Sci (Weinh). 2026 Aug 21:e77306. Online ahead of print. doi: 10.1002/advs.77306 (PMC13496278; doi:10.1002/advs.77306)
Supplement: Supplementary file 1 [file ADVS-9999-e77306-s001.docx]

**Supplementary Materials for**

**Targeting β2-Adrenergic Receptor Stability with Lycorine Reveals a Host-Directed Pathway for Antiviral Defense against Poxviruses**

Xin Zhang^1 #^, Shuqin Xu^2,3 #^, Zixuan Zhang^1#^, Chenwen Wan^1^, Xiaoqin Wang^1^, Peihua Wang^1^, Huini Yang^1^, Tianxiao Huang^3,5^, Yijia Yuan^1^, Faiza Atique^1^, Rui Yang^4*^, Yingli He^1*^, Zhijun Liu^1*^

Corresponding author: Zhijun Liu, zhijun_liu@xjtufh.edu.cn

**Fig S1.** **Chronic stress exacerbates CPXV infection and lethality in vivo. A.** Analysis of serum Corticosterone and Noradrenaline levels in mice with or without CRS treatment by ELISA. **B.** Representative images of mice movement trajectory in elevated plus maze (EPM) in different groups. The EPM was performed on day 8. n = 10 mice per group. **C.** Open arm distance, entries and times, were recorded in EPM in control and FS mice. **D.** Representative images of mice movement trajectory in open field test (OFT) in different groups. The OFT was performed on day 8. n = 10/group. **E.** Center distance, entries and times were recorded in OFT in control and FS mice. **F.** Representative GFP fluorescence images of BMDMs isolated from CPXV infected control and CRS mice. The intensity of GFP signal correlates with the level of viral infection. **G-H.** Measurements of viral proteins (E3L, L1R, D8L, WR117) and genes (A3L, E3L, D13L) expression in BMDMs from mice subjected to CRS followed by CPXV infection (n=3~5/group) by immunoblot or RT-PCR. **I.** Transcript levels of pro-inflammatory cytokines were measured in mouse epididymal fat inoculated with BPL‑inactivated CPXV, with non‑inactivated virus epididymal fat serving as controls. **J.** Measurements of viral proteins expression in BMDMs from mice subjected to FS followed by CPXV infection (n=3/group) by immunoblot. Data are presented as mean ± SEM. n represents the number of biologically independent animals as detailed above. Statistical analyses were determined using unpaired two-tailed Student’s t tests unless otherwise indicated. **p* < 0.05, ** *p* < 0.01, ****p* < 0.001, *****p* < 0.0001; *ns*, not significant.

**Fig S2.** **Alleviation of anxiety like behavior confers protective resistance against viral infection in the host. A.** Experimental design of CPXV infection following enrichment environment (EE). C57 BL/6 male mice fed to EE for 5 weeks. Then, all mice were infected with CPXV for subsequent survival observation or tissue collection. **B.** Representative images of mice movement trajectory in EPM in different groups. n = 10 mice per group. **C.** Open arm distance, entries and times in EPM in the mice of control and EE groups. **D.** Representative images of mice movement trajectory in OFT between control and EE groups. n = 10/group. **E.** Center distance, entries and times in OFT in the mice of control and EE groups. **F.** Viral titers in epididymal adipose tissue, spleen and liver from control and EE mice (n=10/group) at 3.5 dpi. **G.** Survival of control and EE mice following CPXV infection (n=10/group). Statistical significance was determined by the log-rank (Mantel-Cox) test. **H.** Representative GFP fluorescence images of BMDMs isolated from CPXV infected control and EE mice. **I.** Measurements of viral proteins (E3L, L1R, D8L, WR117) expression in BMDMs from infected mice. Data are presented as mean ± SEM. n represents the number of biologically independent animals as detailed above. Statistical analyses were determined using unpaired two-tailed Student’s t tests unless otherwise indicated. **p* < 0.05, ** *p* < 0.01, ****p* < 0.001, *****p* < 0.0001; *ns*, not significant.

**Fig S3. Autophagy-mediated downregulation of** **β2-AR underlies stress-exacerbated viral pathogenesis. A.** β2-AR protein expression in BMDMs from control, CRS, FS and EE groups (n=3/group). **B.** Graphical summary of J2 macrophages infection timeline. **C-E**. Effects of CPXV infection time and MOI on β2-AR expression in J2 macrophages (C) and RAW264.7 (D, E). **F.** Immunostaining assay was used to analyze and quantify the colocalization of β2-AR (red) and CPXV-GFP (green) in RAW264.7. Scale bar:10µm. **G.** Schematic of the procedure for isolating and culturing BMDMs following CPXV infection. **H.** β2-AR mRNA levels in CPXV-infected BMDMs at the indicated time points (n=4~8/group). **I.** β2-AR expression in BMDMs following in vivo CPXV infection. Mice were infected intraperitoneally with CPXV, and BMDMs were isolated at 24, 48, 72, and 96 hours post-infection (n=2/group). **J.** Effects of Bafilomycin A1 on CPXV-induced expression of β2-AR. J2 macrophages cells (1 × 10^6^) were pre-treated with Bafilomycin A1 (0.1μM) for 1h, and cells were uninfected or infected with CPXV (MOI = 0.1) for 24h, and immunoblot analysis and quantitation was performed. **K.** Effects of MG132 on CPXV-induced expression of β2-AR. J2 macrophages cells (1 × 10^6^) were uninfected or infected with CPXV (MOI = 0.1) for 24h and treated with MG132 (10μM) for 8h. Then, immunoblot analysis and quantitation were performed. Data are presented as mean ± SEM. n represents the number of biologically independent animals as detailed above. Statistical analyses were determined using unpaired two-tailed Student’s t tests unless otherwise indicated. **p* < 0.05, ** *p* < 0.01, ****p* < 0.001, *****p* < 0.0001; *ns*, not significant.

**Fig S4. Restoring β2-AR signaling restricts CPXV infection and pathogenesis. A.** Process for Salmeterol treatment in CPXV-infected cells. **B.** Antiviral effects of Salbutamol (10μM,5μM) on CPXV-induced viral replication. **C-D.** Effects of Isoprenaline (D) or Eformoterol Hemifumarate (E) on CPXV-induced viral infection. **E.** Expression of Salmeterol on CPXV infection-induced inflammation. **F.** Schematic of the generation of myeloid-specific knockout mice using CRISPR/Cas9. **G.** Validation of knockout efficiency in myeloid-specific knockout mice by qRT-PCR analysis. **H.** Viral genes expression of BMDMs isolated from CPXV infected β2-AR^f/f^ and β2-AR^Lyz-KO^ mice (n=3/group). Data are presented as mean ± SEM. n represents the number of biologically independent animals as detailed above. Statistical analyses were determined using unpaired two-tailed Student’s t tests unless otherwise indicated. **p* < 0.05, ** *p* < 0.01, ****p* < 0.001, *****p* < 0.0001; *ns*, not significant.

**Fig S5. Identification of lycorine as a β2-AR-stabilizing antiviral compound against CPXV infection. A.** Graphic for lycorine treatment in CPXV or VACV infected cells. **B.** Assessment of the cytotoxicity and antiviral ability of lycorine. The effect of lycorine treatment at different concentrations on cell viability and antiviral ability were determined by CCK-8 assay. The CC50 and EC50 values were determined to be 20.15 μM and 1.07 μM, respectively, by nonlinear regression analysis. **C-D.** Antiviral activity validation of the candidate drugs Amentoflavone (C) and Honokiol (D). **E.** β2-AR-targeting compound lycorine and its potential poly-pharmacological activities from TCM Bank. **F.** Statistical analysis of protein-ligand interactions for β2-AR_L. (Upper) Two-dimensional interaction diagram of residues with interaction frequencies greater than 30%. (Lower) Statistical plot of all amino acids interacting with the ligand molecule. **G.** Interaction frequency and molecular docking results of lycorine with the β2-AR_MOUSE protein. **H.** Immunoblot analysis of the binding between lycorine and β2-AR in Vero cells via DARTS. Data are presented as mean ± SEM. n represents the number of biologically independent animals as detailed above. Statistical analyses were determined using unpaired two-tailed Student’s t tests unless otherwise indicated. **p* < 0.05, ** *p* < 0.01, ****p* < 0.001, *****p* < 0.0001; *ns*, not significant.

**Fig S6. Lycorine exerts post-entry antiviral activity against orthopoxvirus infection. A.** Potency of lycorine against VACV replication in BS-C-1 cells. **B.** Western blot analysis of VACV protein expression (E3L, L1R, D8L, WR117) in BS-C-1 cells infected at increasing MOIs. **C.** Protective effect of lycorine pre-treatment at different time points against CPXV infection. Viral replication was monitored by plaque assay. Data are presented as mean ± SEM. n represents the number of biologically independent animals as detailed above. Statistical analyses were determined using unpaired two-tailed Student’s t tests unless otherwise indicated. **p* < 0.05, ** *p* < 0.01, ****p* < 0.001, *****p* < 0.0001; *ns*, not significant.

**Fig S7.** **Lycorine protects against CPXV infection through myeloid β2-AR signaling and CD8^+^ T cell-dependent immunity. A-B.** Schematic illustration of the experimental design and treatment regimen for lycorine administration in CPXV-infected mice. **C.** Immunoblot analysis and quantification of viral protein (E3L, L1R, D8L, WR117) in liver tissues from CPXV-infected mice treated with vehicle or lycorine (10 or 20mg/kg). **D.** Representative immunofluorescence images of F4/80⁺ macrophages in epididymal adipose tissue. Quantification of F4/80⁺ area per field is shown in the right panel (n=6 fields/group. Scale bars, 100 μm. **E.** Representative immunofluorescence images confirming depletion of CD8⁺ T cells in epididymal adipose tissue. Scale bars, 100 μm. **F.** Representative immunofluorescence images confirming depletion of NK1.1^+^ cells in epididymal adipose tissue. Scale bars, 100 μm. Data are presented as mean ± SEM. n represents the number of biologically independent animals as detailed above. Statistical analyses were determined using unpaired two-tailed Student’s t tests unless otherwise indicated. **p* < 0.05, ** *p* < 0.01, ****p* < 0.001, *****p* < 0.0001; *ns*, not significant.

**Fig. S8 Lycorine suppresses TBK1/IRF3-driven inflammatory signaling while promoting β2-AR-associated IFN/ISG responses. A.** Immunoblot analysis of STING, p-TBK1 and p-IRF3 in BS-C-1 cells infected with VACV at the indicated MOIs and treated with lycorine (2.5μM). **B.** Immunoblot analysis of STING, p-TBK1 and p-IRF3 in BS-C-1 cells infected with VACV (MOI=0.1) at the indicated time points and treated with lycorine (2.5μM). **C.** qRT-PCR analysis of MX1 and OAS2 expression in CPXV-infected BMDMs from β2-AR^f/f^ and β2-AR^Lyz-KO^ mice treated with salmeterol for 24 h (n = 3/group). **D.** qRT-PCR analysis of MX1 and OAS2 expression in CPXV-infected BMDMs from WT and STING^-/-^ mice treated with salmeterol for 24 h (n = 3/group). **E.** Immunoblot analysis of AKT phosphorylation in CPXV-infected cells at the indicated MOIs. **F.** Immunoblot analysis of AKT phosphorylation in VACV-infected cells at the indicated MOIs. **G.** Immunoblot analysis of AKT phosphorylation in VACV-infected cells at the indicated time points. **H.** Effects of the AKT inhibitor AKT IV on CPXV infection, assessed by plaque assay and GFP fluorescence. **I.** Immunoblot analysis of p-AKT in VACV-infected cells at the indicated MOIs and treated with lycorine (2.5μM). **J.** Immunoblot analysis of p-AKT in VACV-infected cells (MOI=0.1) at the indicated time points and treated with lycorine (2.5μM). Data are presented as mean ± SEM. n represents the number of biologically independent animals as detailed above. Statistical analyses were determined using unpaired two-tailed Student’s t tests unless otherwise indicated. **p* < 0.05, ** *p* < 0.01, ****p* < 0.001, *****p* < 0.0001; *ns*, not significant.
